# Stirring the Pot: Can Dietary Modification Alleviate the Burden of CKD?

**DOI:** 10.3390/nu9030265

**Published:** 2017-03-11

**Authors:** Matthew Snelson, Rachel E. Clarke, Melinda T. Coughlan

**Affiliations:** 1 Glycation, Nutrition and Metabolism Laboratory, Baker IDI Heart and Diabetes Institute, Melbourne 3004, Australia; rachel.clarke@bakeridi.edu.au; 2 Department of Physiology, Monash University, Clayton 3800, Australia; 3 Department of Diabetes, Central Clinical School, Monash University, Alfred Medical Research and Education Precinct, Melbourne 3004, Australia

**Keywords:** advanced glycation end products, albuminuria, diet, chronic kidney disease, diabetes, cardiovascular disease, inflammation

## Abstract

Diet is one of the largest modifiable risk factors for chronic kidney disease (CKD)-related death and disability. CKD is largely a progressive disease; however, it is increasingly appreciated that hallmarks of chronic kidney disease such as albuminuria can regress over time. The factors driving albuminuria resolution remain elusive. Since albuminuria is a strong risk factor for GFR loss, modifiable lifestyle factors that lead to an improvement in albuminuria would likely reduce the burden of CKD in high-risk individuals, such as patients with diabetes. Dietary therapy such as protein and sodium restriction has historically been used in the management of CKD. Evidence is emerging to indicate that other nutrients may influence kidney health, either through metabolic or haemodynamic pathways or via the modification of gut homeostasis. This review focuses on the role of diet in the pathogenesis and progression of CKD and discusses the latest findings related to the mechanisms of diet-induced kidney disease. It is possible that optimizing diet quality or restricting dietary intake could be harnessed as an adjunct therapy for CKD prevention or progression in susceptible individuals, thereby reducing the burden of CKD.

## 1. Introduction

Chronic kidney disease (CKD) is a broad term given to a range of disorders characterised by impaired kidney structure and function [1]. Chronic conditions such as diabetes, obesity, hypertension or cardiovascular disease can lead to the development of CKD; however, it can also occur in the absence of disease due to aging, exposure to toxins or infection [1]. The current diagnostic criterion for CKD is a glomerular filtration rate (GFR) of <60 mL/min per 1.73 m^2^ or a urinary albumin to creatinine ratio of >30 mg/g [2]. Based on this definition, CKD is estimated to affect more than 10% of the global population [3]. A GFR of <60 mL/min per 1.73 m^2^ is associated with an increased risk of cardiovascular disease (CVD) mortality and all-cause mortality [4]. Given these observations, CKD is poised to become a major burden to health care systems globally, particularly as populations continue to age.

The kidney plays a central role in maintaining homeostasis in the body, and is often the target organ of inflammatory, metabolic and systemic vascular disorders [5]. Nutrition and dietary patterns are implicated in the development of chronic metabolic diseases, and are modifiable factors that can be utilised to prevent or slow the progression of CKD. The modern Western diet, composed of foods that are high in fat, protein, sugar and sodium and low in fibre, is considered to be a key driver behind the current epidemic of chronic diseases [6,7]. In comparison, balanced diets consisting of moderate fat and high in whole grain carbohydrates are associated with reduced risk of disease and improved mortality [7]. Dietary therapy has historically been used in the management of CKD and guidelines exist surrounding the intake of protein and sodium for patients with CKD [8]. Adherence to current dietary guidelines can reduce the incidence, or slow the progression of CKD and improve mortality [9]. Evidence is emerging to suggest that there are many other nutrients that can potentially influence kidney health either through metabolic or haemodynamic effects, or via the modification of gut homeostasis including changes to gut microbiota. The purpose of this review is to summarise the key recommendations to date, and to provide an overview of recent literature pertaining to potential novel modifiable dietary components that may be useful in the treatment of CKD.

## 2. Protein

Dietary protein restriction has been recommended for individuals with CKD for decades. Early experimental evidence in a number of rodent models of kidney disease, including models of diabetic nephropathy and spontaneously hypertensive rats, consistently demonstrated that low protein diets ameliorate the progression of glomerular dysfunction [10,11]. Evidence that protein restriction was beneficial in improving albuminuria in patients with kidney disease was first described in 1986 [11,12]. Current recommendations for protein intake in individuals with CKD stages 1–4 is 0.8 g/kg body weight, and avoidance of high protein intake (>1.3 g/kg/day) [13]. The key benefits of protein restriction are primarily thought to arise from the amelioration of proteinuria and it has been proposed that an even lower protein intake of 0.6–0.8 g/kg bodyweight in patients with CKD stages 3–5 may be desirable [11,14]. Further dietary protein reduction, using a very low protein diet supplemented with ketoanalogues of essential amino acids (0.35 g/kg/day) has been shown to reduce blood pressure in CKD patients [15,16]. Other factors that occur due to excess protein metabolism, such as increased GFR [17], metabolic acidosis [11] and oxidative stress [18], are also thought to contribute to kidney damage associated with high protein intake [11]. Despite substantial experimental evidence to indicate that dietary protein restriction prevents the development of proteinuria and renal fibrosis [19,20,21], many clinical studies in humans have failed to find the same level of renal protection [11,22,23,24]. The Modification of Diet in Renal Disease (MDRM) study found that a low protein diet (0.58 g protein/kg/day) did not significantly improve glomerular filtration rate in CKD patients when compared to a standard protein diet (1.3 g/kg/day) at a two-year follow-up [24]. This is likely because of the extreme differences in protein content used in experimental diets, but may also be due to the effect of other factors associated with protein foods, such as energy, water, sodium and phosphorous, that may affect kidney function [11]. Protein energy wasting is a major complication of kidney disease [25], particularly in later stages. A long-term follow-up of patients from the MDRM study found that assignment to a very low protein diet supplemented with a mixture of essential keto acids and amino acids (0.28 g protein + 0.28 keto and amino acids/kg/day) was associated with a significantly greater risk of death compared to a low protein diet (0.58 g/kg/day) [26]. Though it was not clear whether this finding may be due to the reduced mean energy intake that occurred with the very low-protein diet, or the possible toxicity of the keto acids and amino acids supplement [26]. Consequently, effective strategies to improve GFR or reduce proteinuria without jeopardising protein balance are a high priority in advancing CKD treatment.

### 2.1. Protein Source and Metabolic Acidosis

**Figure 1:**
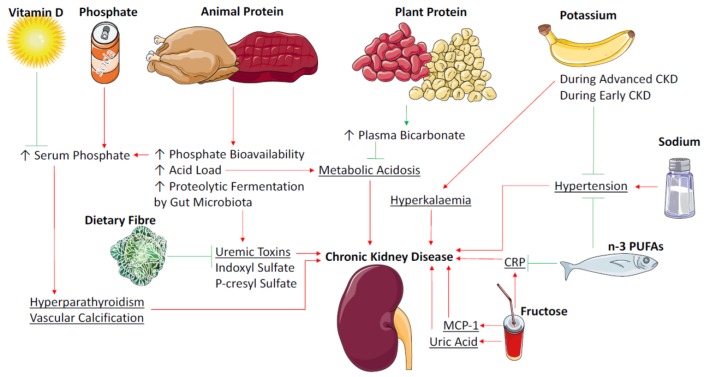
Mechanisms of dietary factors impact on chronic kidney disease. *n*-3 PUFAs = Omega 3 Polyunsaturated Fatty Acids. CRP = C-Reactive Protein. MCP-1 = Monocyte Chemoattractant Protein-1.

Growing evidence suggests that the source of protein (plant or animal) may be more important than the quantity of protein consumed (Figure 1). One reason for this is the tendency for excess meat intake to disrupt the acid-base balance. Metabolic acidosis is a common occurrence in CKD patients and results in low circulating bicarbonate—a risk factor for the progression of nephropathy [27,28] and is associated with increased mortality [29]. Protein from animal sources is composed of sulphur-containing amino acids which, when oxidized, generate sulphate, a non-metabolizable anion that contributes to total body acid load [30]. Protein from plant sources contains higher levels of glutamate, an anionic amino acid that upon metabolism consumes hydrogen ions to remain neutral, thereby reducing acidity levels [30]. Plant foods are also generally higher in anionic potassium salts, which also result in the consumption of hydrogen ions upon metabolism and reduction in acid load [30]. In response to an increase in acid load the kidney adapts by increasing ammonium ion excretion in order to expel excess hydrogen ions, therefore increasing the demand for ammonia production [30]. This stimulates the breakdown of glutamine and other amino acids promoting protein catabolism and muscle wasting [31] while also leading to renal hypertrophy [32]. Metabolic acidosis also promotes protein muscle wasting via the activation of the ATP-dependent ubiquitin-proteasome system [33]. In response to a high acid load, the kidney also undergoes functional changes including promotion of glomerular hyperfiltration and renal vasodilation, features typical of early diabetic kidney disease [30]. Results of the Chronic Renal Insufficiency Cohort Study suggest that consumption of a greater proportion of protein from plant sources is associated with higher bicarbonate levels as well as an improved phosphorous balance in patients with CKD [34]. Trials in humans investigating the effect of plant source protein versus animal source protein are limited, but so far suggest that increasing the proportion of dietary protein from plant foods improves kidney health. A longitudinal controlled trial in patients with diabetic nephropathy reported that replacing proteins from animal sources with soy protein improved proteinuria and urinary creatinine as well as markers of cardiovascular disease [35]. Another study in healthy adults found that intake of vegetable protein, independent of total protein intake reduced glomerular filtration rate, renal plasma flow and improved albumin clearance compared to animal protein [36]. A recent study compared the effect of a very low protein vegetarian diet (0.3 g/kg/day) supplemented with ketoanolouges to a standard mixed-source low protein diet (0.6 g/kg/day) on CKD progression [37]. At an 18-month follow-up significantly fewer patients from the very low protein vegetarian diet group required renal replacement therapy, or reached the primary end-point of >50% reduction in GFR [37]. A study in CKD patients with dual RAAS blockade, noted that high serum phosphate levels attenuated the antiproteinuric effect of a very low protein diet with ketoanalouges [16], and animal protein sources contain more bioavailable phosphate, which not only increases total body acid levels but is also understood to be a uremic toxin that is associated with all-cause mortality (discussed below) [32]. A very low protein vegetarian diet has also been noted to reduce metabolic acidosis in a cohort of CKD patients [38]. Fruit and vegetables are sources of dietary alkali and interventions to increase fruit and vegetables in CKD populations have been shown to increase plasma CO_2_ levels [39,40], indicative of amelioration of metabolic acidosis. Taken together, the evidence suggests that increasing the proportion of plant-based protein intake in patients with CKD may improve renal outcomes, provided there is an adequate quantity of protein to prevent protein energy wasting.

### 2.2. Protein Fermentation by the Colonic Microbiota

The microbial metabolism of protein also produces a number of metabolites that may negatively affect the kidneys. The fermentation of protein in the colon results in the production of indoxyl sulphate and p-cresylsulphate (the conjugated form of p-cresol), which are known nephrotoxic compounds [41]. Circulating indoxyl sulphate can increase oxidative stress in the renal tubular cells and the glomeruli [42]. Also, in vitro indoxyl sulphate has been observed to activate inflammatory pathways resulting in an increase in the expression of monocyte chemoattractant protein-1 (MCP-1) and intracellular adhesion molecule-1 (ICAM-1) [43]. P-cresylsulphate has similarly been linked to CKD and CVD mortality, although the mechanism is not as well defined [44,45]. Interestingly, it has been noted that vegetarians have lower levels of these nephrotoxic compounds compared with omnivores, in both healthy [46] and CKD populations [47]. Vegetarians tend to have higher fibre intakes [46], which could be metabolized by the colonic microbiota instead of amino acids, leading to a reduction in indoxyl sulphate and p-cresylsulphate. This provides another mechanism to explain why vegetarian protein sources appear less detrimental than animal protein sources. Furthermore, carnitine and lecithin present in red meat are metabolized by the microbiota to form trimethylamine-*N*-oxide [48], which has been linked to cardiovascular events. The interaction between animal sources of protein and gut bacteria in CKD warrants further investigation. Determining an optimum protein to fibre ratio could allow for appropriate protein intake to prevent protein energy wasting, without adverse effects on renal outcomes.

## 3. Dietary Fibre/Non Digestible Carbohydrates

Dietary fibre was considered as a treatment for chronic renal failure more than 30 years ago, where it was found to reduce plasma urea [49]. Since then, interest has extended to a variety of non-digestible carbohydrates for their abilities to impact markers of CKD. Non-digestible carbohydrates are resistant to hydrolysis by human digestive enzymes, are able to pass through the gastrointestinal tract into the large intestine and include dietary fibres, non-starch polysaccharides, β-linked oligosaccharides and resistant starch [13].

### Human Studies—Intervention

Chronic kidney disease results in a state of chronic low-grade inflammation, with increases seen in pro-inflammatory markers such as interleukin 6 (IL-6) and C-reactive protein (CRP), which contributes to worsened mortality outcomes in this population [50]. Epidemiological survey data indicated an inverse association between dietary fibre intake and the inflammatory marker CRP and mortality in patients with CKD [51]. Such epidemiological data should be interpreted with caution, however, as there is some uncertainty about whether dietary fibre per se is beneficial or whether other nutrients, including antioxidant compounds that are present in fibre-rich foods, act in a beneficial manner [52]. Interventions that have focused on increasing total dietary fibre intake in patients with pre-dialysis CKD have reported reductions in serum creatinine levels [53] and plasma p-cresol [54]. A four-week study in which patients with chronic renal failure consumed 50 grams per day of acacia gum, a highly fermentable fibre, led to a mean reduction in plasma urea of 12% [55]. Supplementation with acacia gum for three months led to decreases in serum urea, creatinine and phosphate by 31%, 10% and 22%, respectively [56]. A recent meta-analysis of human trials found that dietary supplementation with fermentable fibres was associated with a reduction in serum urea and creatinine in patients with stage 3–5 CKD (pre-dialysis only); however, it should be noted that most of the trials (86%) that were reviewed were considered to be of a low quality [57]. Recently, several short term studies have been undertaken using non digestible carbohydrates in patients receiving dialysis. A four-week Belgian study in haemodialysis patients showed that plasma p-cresylsulphate decreased by 20% when supplemented with oligofructose-enriched inulin [58]. This result has been echoed in a similar study that combined galacto-oligosaccharides with probiotics [59]. Whilst neither of these studies showed a reduction in indoxyl sulphate, a recent six-week dietary intervention with resistant starch in haemodialysis patients led to a mean reduction of plasma indoxyl sulphate and p-cresylsulfate by 29% and 28%, respectively [60]. Whilst these studies show an improvement in the levels of uremic toxins, this has yet to be translated into hard clinical outcomes such as CVD events and mortality.

Current Australian guidelines recommend that patients with early CKD consume a diet rich in dietary fibre; however, this recommendation was given the lowest evidence grading score (2D), indicating that this is a weak recommendation based upon very low-quality evidence [61]. Other guidelines for the management of CKD make no mention to the role of non-digestible carbohydrates [8], which some commentators feel should be rectified on the basis of emerging evidence [52]. Dietary fibre intake is about 20%–30% lower in haemodialysis patients compared to control subjects [62,63], with dialysis patients consuming approximately 11 (±6) g/day dietary fibre, significantly less than the recommendation of 25 g/day [64]. These data suggest that non-digestible carbohydrates are effective at improving biochemistry markers in haemodialysis patients, and that dietary interventions involving these compounds may be particularly relevant given the low intakes seen in this population.

The use of non-digestible carbohydrates for the treatment of CKD is an emerging field and it has been noted that there is a paucity of studies related to clinical outcomes [65]. The use of dietary fibre supplementation is a simple, non-invasive option that does not negatively impact patients’ quality of life [66], although some prebiotic compounds have been noted to have minor negative gastrointestinal effects when consumed at high doses [67].

## 4. Sodium

High dietary sodium intake is a risk factor for hypertension, which is understood to be both a cause and a consequence of CKD [68]. Hypertension promotes glomerular hyper-filtration and proteinuria and therefore increases the rate of progression of CKD [8,69]. High blood pressure can lead to vascular remodelling in the kidney, which is thought to be the cause of subsequent tubular atrophy, glomerulosclerosis and reduced filtration surface area [69]. These structural changes in the kidney, whether they initially occur due to hypertension or by other factors such as diabetes, impair the excretion of sodium [70]. Therefore, high dietary sodium intake in CKD can worsen existing hypertension, or result in the development of salt-sensitive hypertension [68]. Current guidelines for sodium intake for individuals with CKD stages 1–4 are less than 2000 mg per day [13]. These recommendations are based on the results of a large systematic review of experimental and non-experimental studies, which, despite varying quality and heterogeneity of included studies, consistently show that high sodium intake is associated with kidney tissue injury and worsening albuminuria [71]. Salt restriction in patients with moderate to severe CKD has been shown to significantly reduce blood pressure, albuminuria and proteinuria [72]. The degree of improvement in these markers was significantly greater in patients with CKD than without, supporting the idea that patients with CKD are particularly salt-sensitive [72]. Interestingly, restricting dietary sodium intake in patients with CKD on angiotensin converting enzyme (ACE) inhibitors was more effective than dual blockade of the renin-angiotensin-aldosterone system (ACE inhibitor plus an aldosterone receptor blockade) in reducing blood pressure and proteinuria [73]. This trial, although possibly underpowered for a blood pressure study, highlights the importance of CKD patients accomplishing sodium restriction while being treated with ACE inhibitors to best improve renal markers.

## 5. Potassium

In addition to the direct benefits of reducing dietary sodium intake on blood pressure and proteinuria, reducing consumption of high sodium foods generally increases the amount of potassium in the diet [74]. Potassium is understood to be antihypertensive and may abolish sodium sensitivity [74]. An improved sodium to potassium ratio may be one reason why the Dietary Approaches to Stop Hypertension (DASH) diet, which contains twice the potassium of a standard western diet, is effective in reducing blood pressure [74]. The DASH diet has not been widely assessed in patients with CKD due to the high protein, potassium, calcium and phosphorous content [75]. In patients with severe CKD, such as those on dialysis, impaired potassium excretion leads to hyperkalaemia, which is associated with higher all-cause mortality [76]. For this reason intake of potassium is restricted in these patients. However, during earlier stages of CKD a diet high in potassium, such as a diet rich in fruits and vegetables, may slow progression to later stages through lowering blood pressure [74]. A small retrospective study found that the DASH diet was still effective in individuals with reduced kidney function at baseline [75]. Larger studies are required before the DASH diet can be recommended to CKD patients. However, it is likely that the benefits of plant-based diets naturally high in potassium and fibre and low in acidogenic proteins and minerals would outweigh the potential risk of developing hyperkalaemia in early CKD. In a group of stage 4 CKD patients, selected to be at low risk for hyperkalaemia, treating metabolic acidosis with base-producing vegetables was effective in improving metabolic acidosis and reducing kidney injury [40]. Larger long-term trials in CKD patients investigating plant-based diets on renal biomarkers and clinical outcomes are warranted on the basis of the positive findings to date.

## 6. Vitamin D

The kidneys play an important role in the metabolism of vitamin D into its active form, from vitamin D precursors which are obtained either through the diet or from conversion of 7-dehydrocholesterol in the skin by UV light. These precursors are converted in the liver to calcidiol (25 hydroxy vitamin D), which is further converted into the active form of vitamin D, calcitriol (1,25-dihydroxy vitamin D3) in the mitochondria of the proximal convoluted tubules of the kidney, by an enzyme called renal 1-α hydroxylase. As kidney function declines there is a direct decrease in the synthesis of calcitriol [77]. Calcitriol suppresses the release of parathyroid hormone (PTH); however, in CKD this mechanism is blunted due to decreased production of calcitriol, leading to over-release of parathyroid hormone in a condition called secondary hyperparathyroidism. This secondary hyperparathyroidism can lead to alterations in bone turnover and metabolism and the development of renal osteodystrophy [78]. Vitamin D deficiency and secondary hyperparathyroidism are recognised to be complications associated with chronic kidney disease [61].

### 6.1. Low Vitamin D, CKD and Association with Mortality

Cardiovascular disease is a significant contributor to mortality in patients with renal disease, with sudden cardiac death accounting for 20%–30% of deaths in dialysis patients [79]. Haemodialysis patients with a severe vitamin D deficiency (≤25 nmol/L of 25(OH)D) have a threefold higher risk of sudden cardiac death compared with those with vitamin D levels greater than 75 nmol/L [80]. A meta-analysis of data from observational studies showed that, for each 25 nmol/L increase in serum levels of 25(OH)D there was a significant decrease in the relative risk (RR = 0.86, CI: 0.81–0.92) of mortality [81]. Altered vitamin D levels and subsequent hyperparathyroidism can contribute to the formation of extracellular insoluble calcium phosphate and subsequent calcification of the vasculature [78]. Coronary artery calcification has been reported in patients with CKD [82], and calcium phosphate levels have been shown to correlate with increased mortality risk in HD patients [83]. Low plasma 25(OH)D is also associated with a higher risk of developing increased albuminuria, particularly in individuals with high sodium intake [84]. Thus it is thought that correcting vitamin D levels may reduce PTH levels, correct alterations in bone turnover and calcium metabolism, and subsequently reduce mortality in the CKD population.

### 6.2. Vitamin D Supplementation and Parathyroid Hormone

Newer vitamin D analogues, such as paricalcitol, play an important role in CKD as they appear to have better suppression of parathyroid hormone and possibly less of a calcaemic effect compared to other vitamin D sterol forms [85]. Several studies have shown that paricalcitol supplementation in CKD patients was associated with a decrease in PTH levels [86,87,88]. The study by Alborzi et al. did not find any change in PTH levels, which may be due to its shorter duration of one month [89]. Data from both observational studies and RCTs showed that vitamin D supplementation improves levels of parathyroid hormone in both pre-dialysis and dialysis-requiring patients [90]. Whilst it may be effective in lowering parathyroid hormone levels, some concern has been raised over the potential risk that paricalcitol may exacerbate vascular calcification [91].

### 6.3. Vitamin D Supplementation and Proteinuria

Reduction of residual proteinuria is associated with reductions in serum creatinine levels, progression to end-stage renal disease and mortality [92]. Several studies have shown that oral supplementation with the vitamin D analog paricalcitol is effective at reducing proteinuria in stage 2–4 CKD patients [87,88,89,93]. Furthermore, oral paricalcitol therapy achieves these reductions in proteinuria without an increase in adverse events [77]. A meta-analysis of trials in CKD patients showed that supplementation with vitamin D was associated with a mean reduction in proteinuria of 16%, which was a reduction seen in addition to the effect seen by RAS blockade [94]. Whilst vitamin D supplementation does reduce proteinuria, this is not associated with changes in other markers of renal functions, such as GFR, and does not appear to alter the risk of pre-dialysis CKD patients progressing to dialysis [85,91]. This lack of an effect on clinical outcomes is perplexing, as trials that have used RAS blockers to reduce proteinuria to a similar extent were associated with improvements in GFR and reduced progression to ESRD [92,95,96]. A possible explanation may be insufficient study duration; in the meta-analysis by Xu et al. 12 studies assessed vitamin D supplementation and GFR, of which seven had an intervention that lasted for six months or less; three studies ran for 12 months, whilst the remaining studies had durations of 18 and 24 months, respectively.

### 6.4. Vitamin D Supplementation and Clinical Outcomes—Mortality

Whilst vitamin D supplementation has been shown to alter biochemical parameters of patients with CKD, the effect on morbidity and mortality outcomes in this patient group is less clear. A meta-analysis of observational studies showed that patients with CKD receiving vitamin D supplementation had a reduction in risk of all-cause mortality and cardiovascular mortality [97]. However, a recent meta-analysis that looked specifically at RCTs that assessed the effect of vitamin D supplementation on all-cause and cardiovascular mortality in CKD patients found no evidence that supplementation affected mortality outcomes [98]. Of the patients in this meta-analysis, about two thirds were followed up for less than a year, and it has been suggested that this may be insufficient follow-up time to capture CVD events, which are a major contributor to mortality in the CKD population [99].

Many studies assessing the efficacy of vitamin D in CKD patients utilise biochemical outcomes, such as parathyroid levels or proteinuria, rather than clinical endpoints such as progression to ESRD or mortality [77]. A recent umbrella review found that there was a lack of convincing evidence for vitamin D supplementation across a range of health outcomes, including chronic kidney disease [100]. Current Australian guidelines recommend vitamin D supplementation in those with early chronic kidney disease and secondary hyperparathyroidism though admits evidence does not exist to support that this leads to improvement in patient-level outcomes [61]. Thus whilst vitamin D may effectively alter biochemical parameters, larger, longer randomised control trials are urgently required to see whether these translate into meaningful patient-centred outcomes.

## 7. Phosphorus

The kidneys play a major role in phosphorus homeostasis with the glomeruli filtering between 3700 and 6100 mg of phosphorus per day, although 75%–85% of this is reabsorbed, primarily through the proximal tubules, resulting in net excretion of between 600 and 1500 mg of phosphorus per day [101]. As kidney function declines, there is a decrease in the number of functioning nephrons and subsequent decrease in phosphorus excretion [102]. As renal function decreases to less than 80% of normal, phosphorus absorption can exceed the rate of clearance by the kidneys, and a subsequent rise in serum phosphate levels is seen [61,103]. Phosphate anions can combine with extracellular cationic calcium to form insoluble calcium phosphate and subsequent calcification can occur, particularly in the cardiovascular system [78]. Hyperphosphataemia is associated with vascular calcification [104], increased cardiovascular disease risk [105] and increased mortality in both predialytic CKD patients [106,107,108] and patients receiving dialysis [109,110,111]. Furthermore several studies have shown that elevated phosphate levels are associated with a faster rate of renal disease progression in CKD patients [112,113,114,115] and healthy subjects with normal renal function [116]. Maintaining normal phosphate levels or minimizing hyperphosphataemia is seen as a crucial step to limit mortality in CKD patients.

### 7.1. Dietary Sources of Organic Phosphorus

Phosphorous may be present in the diet as organic or inorganic phosphate. Protein-rich foods such as legumes, meat, poultry, fish, eggs and dairy products are the main sources of organic phosphate and there is a correlation between dietary intakes of protein and phosphorous [117]; however, a high protein (and high phosphorus) diet does not always translate to increased serum phosphate levels [118]. The bioavailability of organic phosphate varies depending on the food source with plant sources having a limited bioavailability due to the phosphorous being present largely as phytate. Humans (and other monogastric animals) lack the enzyme phytase and thus cannot digest phytate, although some degradation may occur via the intestinal microbiota [119]. Dairy products have about 30%–60% bioavailability, and the highest bioavailability of organic phosphate, in meat products, may be as high as 80% [102]. This difference in phosphorus bioavailability between meat and plant protein sources, may partially explain the benefits of consuming a greater proportion of protein from plants sources, as described above. Phosphate absorption is linearly related to phosphate intake, with bioavailability being the major determining factor in phosphate uptake from the diet [120]. Thus, for organic phosphate, food choices can make a significant difference in the amount of phosphate that is absorbed from the diet.

### 7.2. Dietary Sources of Inorganic Phosphorous

Phosphorous may be added to foods in the form of inorganic additives, which are typically used to improve taste, texture, shelf life or processing time [121]. These additives are primarily inorganic phosphate salts that require no enzymatic digestion and dissociate rapidly in the low pH environment of the stomach; thus inorganic phosphate additives have a high bioavailability of 90%–100% [102]. Phosphoric acid, which is present in cola drinks, has a bioavailability of 100% [121]. Inorganic phosphate additives are found in many processed foods including frozen meals, snack bars, French fries, spreadable cheeses, instant food products and beverages such as sodas, flavoured water, juices and sport drinks [122]. The phosphorous content in a typical Western diet has increased substantially during the past few decades [123] and in many countries dietary phosphorus exceeds the recommended daily allowance [124]. A recent Australian study found that phosphate additives were present in 44% of the most commonly purchased grocery foods, and were particularly prevalent in small goods (96%), bakery products (93%) and frozen meals (75%) [125]. Inorganic phosphate is readily absorbed and has become highly prevalent in the food supply due to the rise of convenience foods and beverages, and is a significant contributor to dietary phosphate load in a typical Western diet.

### 7.3. Reducing Dietary Phosphorus and Serum Phosphate Levels

Given the deleterious effects of hyperphosphataemia and the rising phosphate content of foods, several studies have addressed reducing dietary phosphorus to reduce serum phosphate levels. One small study found that replacing natural protein sources with a low phosphorus protein concentrate can reduce serum levels of phosphate and parathyroid hormone [126]. A randomised controlled trial using dietary education to limit intake of foods containing phosphate additives led to a reduction in serum phosphate levels of 0.6 mg/dL (95% CI: 0.1–1.0 mg/dL), a decrease that, the authors state, corresponds with a 5%–15% reduction in relative mortality risk, based on findings from observational studies [127]. Dietary reduction of phosphate can reduce serum levels; however, whether this translates to clinical benefits is not clear as limitation of dietary phosphate may excessively limit other nutrients—particularly protein, which often correlates with phosphorous intake. A large retrospective cohort study that considered 30,000 haemodialysis patients found that the relationship between serum phosphate levels and mortality is a J-shaped curve and that those patients who had high phosphate levels and high protein intake had lower mortality compared to those patients with high phosphate levels and low protein intake [128]. Dialysis patients on a phosphate-restricted diet have greater mortality than those without phosphate restriction [129], and it has been suggested that excessive phosphate restriction may be associated with decreased dietary protein intake and subsequent protein energy malnutrition, which leads to increased risk of mortality [130]. The conclusion born from these studies is that haemodialysis patients should aim to minimize phosphorous intake whilst not compromising the adequacy of protein intake.

### 7.4. Phosphate to Protein Ratio

This has led to recommendations for using a ratio between the phosphate content and protein content to identify foods that will provide adequate protein whilst properly controlling dietary phosphate [122]. An observational prospective five year study found that haemodialysis patients with higher dietary phosphate:protein ratio had increased mortality, even after serum phosphate levels were controlled for [117]. Egg white has one of the lowest phosphate:protein ratios [102] whilst many processed foods and beverages are high in phosphate with low protein. One study compared the phosphate:protein ratio in meat products prepared with and without phosphate additives found that there was a 60% increase in the phosphate:protein ratio in those products containing additives [131]. Previous studies have shown that phosphate additives to meat and poultry products can lead to a nearly doubling of the phosphorous content of these products [132]. Worryingly whether meats have been enhanced with inorganic phosphate additives, or to what level, may not be easily determined from the nutrition information panel.

Whilst many studies have shown an association between serum phosphate levels and risk of CVD death in CKD patients, it has been noted that there is a dearth of randomised controlled trials with an intervention that modifies dietary phosphate and assesses mortality as an outcome [123]. Whilst this evidence may be lacking for mortality outcomes, the current guidelines recommend maintaining serum phosphate within a normal range for CKD stages 3–5 and dialysis patients, by dietary restriction and the use of phosphate binders [14], with insufficient evidence for recommending dietary phosphate restriction for early CKD patients (stages 1–3) [61]. The current evidence suggests that limiting foods that have a high phosphate:protein ratio (such as spreadable cheeses and egg yolk) and avoiding inorganic phosphate additives (such as those in cola) may improve outcomes for CKD patients. Treating hyperphosphataemia by limiting the intake of protein-rich foods may contribute to mortality in CKD patients.

## 8. Omega-3 Polyunsaturated Fatty Acids (*n*-3 PUFAs)

In the general population fish intake is associated with a reduction in all-cause mortality, which has been attributed to the high content of *n*-3 polyunsaturated fatty acids (*n*-3 PUFAs) [133]. The anti-inflammatory, anti-hyperlipidaemic and antihypertensive effects of *n*-3 PUFAs are well established in the general population [134]; however, there is less conclusive evidence for those patients with CKD. In particular, there is a dearth of conclusive studies within CKD populations with regards to mortality [135,136].

### 8.1. n-3 PUFAs and Triglyceride Levels

Lipid abnormalities may be a common contributing factor to cardiovascular mortality in end-stage renal disease, with elevated triglyceride levels being the major lipid alteration [136]. A 2009 meta-analysis of trials in hyperlipidaemic patients without renal impairment showed that *n*-3 PUFA supplementation has a clinically significant dose-dependent reduction of triglycerides levels [137]. In CKD patients, several studies have found an 8–12-week intervention with daily eicosapentaenoic acid (EPA) and docosahexaenoic acid (DHA) supplementation resulted in decreases in triglycerides [138,139,140,141,142,143,144,145]. Furthermore several studies have shown that these interventions can improve HDL levels [139,140,146,147]. The evidence from these trials suggests that daily *n*-3 PUFA supplementation is effective at ameliorating dyslipidaemia in CKD patients.

However, not all studies have confirmed this effect. A four-week crossover study where patients received 960 mg/day EPA and 620 mg/day DHA found no effect on serum cholesterol or triglyceride levels [148]. Donnelly et al. conducted a four-week crossover study with 3.6 g/day *n*-3 PUFA and saw a non-significant decrease in triglycerides, though there was no washout period between treatments, which may have led to a carryover effect that may have masked the result in the group that received the treatment before placebo [149]. A three-month study found that supplementation with 4 g/day fish oil tended to decrease serum triglycerides; however, this failed to achieve statistical significance (*p* = 0.07) [150]. Longer studies have cast additional doubt on the efficacy of *n*-3 PUFAs to ameliorate dyslipidaemia in CKD patients, with a six-month study in dialysis patients receiving 960 mg/day EPA and 600 mg/day DHA having no effect on triglyceride levels, although this study did see an increase in both HDL and LDL levels [151]. Several other intervention studies with 2–4 g/day fish oil have failed to see any changes in lipid levels after two months [152,153], six months [154,155] or 12 months [156] of treatment. This lack of an effect, particularly in trials of longer duration, has cast some doubt on the efficacy of *n*-3 PUFAs in reducing hyperlipidaemia.

The variation in results observed in intervention studies may result from the small sample sizes, short durations and differences in *n*-3 PUFA dosing regimes [157]. In a recent meta-analysis, subgroup analysis found that the TG lowering effect was greater in patients with higher baseline TG levels [158], which may account for the variation seen in results from these smaller trials. In the trial by Taziki et al. that found reduction in LDL cholesterol and increase in HDL cholesterol after a 12-week intervention with 2 g/day *n*-3 PUFAs, one of the inclusion criteria was hyperlipidaemia with no current lipid lowering medications [147]. Thus positive results are more likely to be seen in studies with patients who had greater degrees of hyperlipidaemia at baseline. The absorption of *n*-3 PUFAs may be increased up to 3-fold between being taken concomitantly with a high fat meal compared with a low fat meal [159] and timing of dosing and concomitant food intake may affect absorption and subsequent effect of the intervention. The majority of studies instructed patients to consume their regular diet, and it has been noted that the increasing prevalence of functional foods fortified with *n*-3 PUFAs may dilute the effect of these intervention trials [160]. Promisingly, a recent meta-analysis that did subgroup analysis that looked at doses of less than 2 g per day found that this more physiologically relevant dosing was able to significantly decrease TG and increase HDL levels [161]. A 2016 meta-analysis confirmed that *n*-3 PUFAs are able to lower TG levels in HD patients; however, no effect was seen on total cholesterol or LDL cholesterol levels [162]. Taken together, the evidence suggests that daily *n*-3 PUFA supplementation is effective at reducing triglyceride levels in CKD patients with hyperlipidaemia.

### 8.2. n-3 PUFAs and Blood Pressure

CKD leads to the development of hypertension, which itself can contribute to the progression of CKD [163], and thus interventions that reduce blood pressure in CKD patients are required. In the context of CKD, several studies have found that an eight-week intervention with 1840 mg EPA and 1520 mg DHA per day resulted in decreases in blood pressure in patients with CKD stages 3–4 [138]. In contrast to the study by Mori et al. [138], several studies of similar duration and intervention found no change in blood pressure in pre-dialysis CRF patients [139] or diabetic patients with proteinuria [164]. In patients with stage 5 CKD undergoing haemodialysis, interventions with 3.6–4 g/day fish oil led to reductions in blood pressure after three months [150] and 12 months [156], but not after one month [149]. A recent meta-analysis of trials in non-CKD populations found that 2 g/day or more EPA+DHA are effective at reducing blood pressure [165] and, whilst the evidence for a benefit of *n*-3 PUFA supplementation on hypertension within a CKD population has not been demonstrated conclusively, high doses (i.e., over 3 g/day) are likely to result in a modest reduction in blood pressure [166].

### 8.3. n-3 PUFAs and Inflammation in HD Patients

CKD patients experience a state of chronic low grade inflammation, which can contribute to disease progression [50]. *n*-3 PUFAs are able to exert an anti-inflammatory effect, because they compete with *n*-6 PUFAs during eicosanoid synthesis [167]. Noori et al. found that a higher *n*-6:*n*-3 PUFA ratio was associated with increased mortality and inflammation, and thus supplementation with *n*-3 PUFAs may exert an effect by shunting production to less inflammatory eicosanoids [168]. A four-week crossover study found a trend towards lower CRP levels in those patients treated with *n*-3 PUFAs, and the failure to achieve significance may be due to a lack of statistical power or the short duration of the study [148]. Similarly, a 12-week intervention study in PD patients found a trend towards lower CRP; however, the small sample size (*n* = 7) may have prevented this result from reaching significance [169]. Saifullah et al. provided HD patients with 2 g/day fish oil for 12 weeks, providing approximately 854 mg EPA and 488 mg DHA per day, and found a reduction in CRP levels [145]. A six-month study conducted by Bowden et al. found daily intervention with 960 mg of EPA and 600 mg of DHA was able to reduce CRP levels [155]. An 8 week study providing 2.4 g fish oil per day (with an EPA:DHA of 2:1) to HD patients with metabolic syndrome found reductions in CRP, TNF-alpha and IL-6 levels; however, the lack of placebo control should be noted [153]. Several recent studies of 3–4 months in duration have failed to see changes in CRP, TNF-alpha or IL-6 levels; however, this may have been due to the small sample sizes [170,171]. A 2016 meta-analysis of *n*-3 PUFA intervention trials in HD patients found that CRP was significantly reduced; however, no effects were seen with other inflammatory markers (IL-6 and TNF-alpha) [162]. The conclusion that intervention with *n*-3 PUFAs are able to lower CRP levels has clinical relevance as serum CRP levels are able to independently predict cardiovascular mortality in patients undergoing haemodialysis [172] or peritoneal dialysis [173].

### 8.4. n-3 PUFAs and Proteinuria/GFR in CKD

An observational, epidemiological study in a Swedish type 1 diabetic population indicated that people whose diets had a greater amount of fish protein had a lower risk of developing microalbuminuria [174]. A 2009 meta-analysis found a pooled effect of a significant reduction on proteinuria following supplementation with *n*-3 PUFA, though the authors noted that many trials consisted of small numbers of patients and therefore may have been underpowered [175]. A 2012 meta-analysis found that in patients with IgA nephropathy, *n*-3 PUFAs did reduce proteinuria; however, this did not translate to any functional improvements, as measured by GFR [176]. A recent crossover randomised control trial in diabetic patients with early signs of kidney dysfunction (CKD Stage 2), found that six weeks of treatment of 4 g/day *n*-3 PUFA led to a reduction of 16% in urinary neutrophil gelatinase-associated lipocalin (NGAL), a marker of kidney dysfunction [164]. However, this study failed to see changes in the classical hallmarks of kidney disease, such as albuminuria. Interestingly, when subgroup analysis was undertaken and results were stratified for RAAS inhibitor use, significant reductions were observed in several urinary markers including NGAL (−27%) and albuminuria (−17%) [164]. A 2015 study found that three months’ intervention with 3 g/day *n*-3 PUFAs did not impact on proteinuria in patients with diabetic nephropathy; however, all patients were currently controlling blood pressure with either an ACEi or ARB, which may have limited the additional benefit of the intervention [177]. An earlier study in type 1 diabetic patients who were not controlling blood pressure found that 900 mg/day EPA reduced albuminuria after three months of treatment, an effect that was sustained up to 12 months of treatment [178]. There is some evidence to suggest that *n*-3 PUFA supplementation may slow proteinuria in those CKD patients with uncontrolled blood pressure; however, this has not been conclusively proven.

### 8.5. Membrane Levels of n3/n6 PUFAs Associated with Mortality Outcomes in HD

Patients with kidney disease have lower serum and erythrocyte plasma membrane levels of *n*-3 PUFAs and higher levels of the *n*-6 PUFA arachidonic acid, compared with controls [146,179]. A retrospective study found a trend between a erythrocyte omega 3 index levels and survival amongst HD patients; however, the fact that this did not reach statistical significance may be ascribed to an insufficient cohort size to achieve adequate statistical power [180]. Recent longitudinal observational cohort studies have found that the DHA content of erythrocyte plasma membranes is able to independently predict mortality outcomes in HD patients [181,182]. Other studies investigating the fatty acid profile of the serum and mortality in HD patients may be predicted by serum DHA levels [183] or the ratio between *n*-3 and *n*-6 [184]. Importantly, the membrane content of erythrocytes is a good indicator of myocardiocyte fatty acid levels [185], which may explain the association between erythrocyte FA levels and cardiovascular morbidity. Higher fish intake is associated with a higher erythrocyte *n*-3 PUFA content [180] and supplementation with *n*-3 PUFAs was able to increase the omega 3 index and *n*-3/*n*-6 ratio in erythrocyte membrane lipids, whilst also decreasing the percentage of saturated lipids that formed part of the membrane in dialysis patients receiving treatment for three [169] or six months [154]. The evidence of these associations, as well as the ability to alter the plasma membrane levels via dietary intervention as explained above has made *n*-3 PUFAs a promising potential dietary therapy for the reduction of mortality in HD patients.

### 8.6. CVD Events and Mortality Outcomes

Whilst fish consumption is associated with a reduction in mortality outcomes in the general population [133], the evidence is less clear in CKD populations. An observational study showed that dietary fish consumption is an independent predictor of survival in haemodialysis patients [186]. In a long-term observational study with a three-year follow-up, HD patients receiving 1.8 g/day EPA had decreased mortality compared to those who did not receive the dietary supplement [187]. Whilst there are many randomised placebo control trials that have assessed biochemical parameters, there has been a paucity of studies that have looked at mortality outcomes. One study in 206 Danish HD patients who had a history of cardiovascular disease found that dietary intervention with 1.7 g/day *n*-3 PUFAs had no effects on mortality but did reduce the risk of a myocardial infarction by 70% [188]. A 2012 study with 203 patients found that daily intervention with 4 g/day fish oil (1600 mg EPA, 800 mg DHA) was associated with higher cardiovascular event free survival [156]. Whilst intervention with fish oil leads to a reduction in cardiovascular events, this has not been shown to alter mortality; however, this may be due to a lack of sufficiently powered studies assessing mortality as a primary outcome [162].

A diet that is high in *n*-3 PUFAs represents a promising avenue for the reduction of proteinuria, dyslipidaemia and cardiovascular events in CKD patients, although whether this translates to reductions in mortality remains unclear. CKD patients do not consume adequate dietary *n*-3 PUFAs [145]. Retention rates in clinical trials using fish oil pill interventions have a median of 97% [175] and there is good adherence to fish oil supplementation [189], indicating that this is a strategy that could be implemented in patients. However, Shapiro et al. [134] recommend that the focus should be on increasing oily fish intake, rather than prescription of fish oil supplements, for cardioprotection in a population with diabetic nephropathy. A recent meta-analysis indicated that fish intake is more protective than fish oil capsules against cerebrovascular disease [190]. This may be due to the fact that *n*-3 PUFAs from fish are more easily absorbed and incorporated into plasma lipids than from fish oil supplements [191] a synergistic effect with vitamins, trace elements or amino acids present in fish [190] or even a ‘protein substitution’ effect, where fish may replace more ‘detrimental’ protein sources, such as processed red meat [192].

## 9. Sugars and Sugar-Derived Products

### 9.1. Dietary Fructose

Excess fructose consumption, such as that found in table sugar (sucrose) or high-fructose corn syrup, is considered to promote features of metabolic syndrome, including insulin resistance, dyslipidaemia, and hypertension—factors that are also associated with an increased risk of CKD [193,194]. In addition, the metabolism of fructose promotes the production of uric acid, a known risk factor for the development and progression of CKD [193,195]. The metabolism of fructose may also directly induce kidney injury via production of reactive oxygen species and chemokines in proximal tubule cells [196,197]. Dietary fructose is widely prevalent in the Western diet, mainly due to added sugars in foods and beverages and may be another modifiable dietary factor in the treatment of CKD. Cross-sectional evidence suggests that consumption of sugary soda, often sweetened with high-fructose corn syrup, is associated with albuminuria [198], serum uric acid [199,200], and a doubling of the risk of CKD [201] in healthy populations. Longitudinal studies, however, have found conflicting results. A study performed in the USA by Bomback et al. failed to observe an association between consuming more than one soft drink per day and the incidence of hyperuricaemia or CKD over a follow-up period of nine years [195]. In contrast, a recent study by Yuzbashian et al. found an increase in the incidence of CKD over a three-year period in a Middle Eastern population who drank more than four sugar sweetened beverages per week compared with those who drank less than 0.5 per week [202]. In this study, sugar-sweetened beverages were taken as the sum of all types of sugar-sweetened soft drinks and fruit juices. When fruit juice intake was considered alone there was no association with CKD [202], suggesting that there could be an additional factor in soft drinks contributing to disease burden. Interestingly, the Nurse’s Health Study found that the consumption of more than two servings per day of artificially sweetened soft drinks was associated with a two-fold increase in GFR decline in women over an 11-year follow-up, with no association found for sugar-sweetened beverages [203]. Clearly, the differences in populations and periods of follow-up, and also differences in the ingredients present in the soft drinks (due to years of production and specific regional preferences) make it difficult to compare these studies. The results of a meta-analysis of epidemiological studies suggest that sugar-sweetened soft drinks, but not artificially sweetened soft drinks, increase the incidence of CKD [204]. There are many possible confounding factors that may affect the results of epidemiological dietary studies. For example, previous history of sugary drink consumption and weight gain could lead to consumption of diet soft drinks, which could explain the unexpected result seen in the Nurse’s Health Study [205]. There are also added sugars present in commonly consumed foods, which could complicate studies that only consider sugar-sweetened beverage consumption. To better understand the relationship between sugar consumption and kidney disease, it is important to also consider experimental evidence and results from dietary interventions.

Experimental studies have shown a direct mechanism for fructose-induced renal injury. Fructose is taken up into cells by the glucose transporters GLUT 5 and/or GLUT2, both of which are expressed in the proximal tubules of the kidney [197]. All fructose entering the cell is rapidly phosphorylated by fructokinase. This rapid phosphorylation can lead to depletion of ATP. This promotes the production of uric acid through purine metabolism pathways and generates reactive oxygen species [197]. The metabolism of fructose via fructokinase in human proximal tubule cells has been shown to increase levels of the pro-inflammatory chemokine Monocyte Chemoattractant Protein (MCP-1) [197]. Consistent with this, studies in healthy rodents have shown that consumption of a high-fructose diet for six weeks resulted in renal hypertrophy and tubulointerstitial injury, in particular cell proliferation and hyperplasia of proximal tubules [196]. In rodent remnant kidney models, fructose at 60% of total diet accelerated glomerulosclerosis, worsened renal function and led to an increase in levels of MCP-1, an effect not seen with a 60% glucose diet [206]. However, whilst animal models are useful in delineating the mechanisms by which fructose may cause kidney damage, rodents have an active enzyme, uricase, absent in humans, that metabolises uric acid [207]. Therefore, rodents require large doses of fructose or inhibition of uricase for an effect to be observed [207]. Thus, human interventions are essential for fully understanding the consequences of high fructose intake.

A systematic review of controlled feeding trials in people with and without diabetes found that replacing other carbohydrates for fructose isocalorically did not affect serum uric acid [208]. However, when fructose was given at 35% excess energy to non-diabetic males there was a significant increase in serum uric acid levels [208]. This suggests that fructose given in excess of total energy requirements will increase serum uric acid levels, while simply replacing other sugars with fructose may not. There have been limited studies that have investigated fructose consumption in patients with chronic kidney disease [209]. In a pilot study by Brymora et al., patients with stage 2 and 3 CKD followed a low-fructose (12 g/day) diet for six weeks, followed by their normal diet, relatively higher in fructose (60 g/day), for six weeks. No effects on glomerular filtration rate, proteinuria, serum or urinary uric acid were observed. However, hsCRP and soluble ICAM-1 were reduced significantly following the diet, and the reduction in ICAM-1 was maintained during return to the regular diet. This suggests that there may be some benefit to reducing dietary fructose intake in CKD patients, though this comes through a reduction in inflammatory markers rather than uric acids level. Further controlled trials in humans are required to determine the relationship between dietary fructose intake and serum uric acid levels to determine whether it may be contributing to this risk factor for CKD, and whether there is a safe level of fructose to consume.

### 9.2. Dietary Advanced Glycation End Products

Advanced glycation end products (AGEs) are compounds that form through a non-enzymatic reaction between a sugar, such as glucose and an amino acid [210]. Diet-derived AGEs form within foodstuffs during the heat treatment of food products, such as processed cereal products, snack foods and foods cooked at high temperatures. The modern Western diet is high in AGEs as many convenience or snack foods undergo heat treatment to improve the flavour and shelf life of a product [210]. Circulating AGEs consist of those endogenously formed, such as under conditions of oxidative stress and hyperglycaemia, and those that come from exogenous sources including foods and tobacco smoke. Elevated levels of circulating AGEs have been observed in both diabetic and non-diabetic CKD patients [211,212,213]. This is likely due to increased endogenous formation [214] as well as reduced renal excretion [215]. Dietary AGEs correlate with serum carboxymethyl lysine (CML) in CKD patients—a well characterised AGE often used as a marker of AGE levels [216]. Elevated serum CML levels were observed to be associated with estimated GFR and CKD, independent of diabetes, in a cross-section study of men and women suggesting CML levels may be a biomarker of CKD [217]. AGEs may be particularly toxic to the kidney through accumulation in renal tissue and/or activation of pro-inflammatory pathways [218,219,220]. AGEs can promote intracellular oxidative stress in mesangial cells and activate transcription factors that promote the expansion of the mesangial matrix (a hallmark of diabetic kidney disease) and are therefore thought to contribute to the renal dysfunction observed in diabetes [221]. AGEs also bind to cellular receptors such as the multi-ligand receptor for advanced glycation end products (RAGE). Upon binding with its ligands, RAGE activates the pro-inflammatory transcription factor nuclear factor kappa-B (NFκB), leading to upregulation of pro-inflammatory cytokines and mediators of vasoconstriction, coagulation and fibrosis [218,220,222]. RAGE is highly expressed on immune cells and therefore its activation can increase systemic levels of inflammation [220]. RAGE is also highly expressed in the kidney and activation of RAGE has been implicated in the pathogenesis of both diabetic and non-diabetic kidney disease [218,220]. AGE ligation with RAGE mediates mitochondrial superoxide production and diabetic nephropathy [223]. In individuals with CKD, increased expression of RAGE has been observed on monocytes and this was found to be inversely associated with GFR and strongly correlated with circulating AGEs [224].

Whilst increased AGEs may be a feature of CKD, it is not yet understood whether this predicts clinical outcomes. The majority of observational studies that have looked at circulating AGEs have found no association between serum CML or pentosidine and cardiovascular events or mortality in patients with either diabetic or non-diabetic kidney disease [225,226,227,228,229]. However, the better nutritional status of patients in one of these studies could explain their increased survival [227]. So far there has been a single study that reported an association between elevated serum levels of CML and all-cause mortality in patients on long-term dialysis [230]. However, it is important to note that patients in this study with higher CML levels had reduced urinary volume and had longer on dialysis, factors that could contribute to reduced survival. These studies do not exclude the possibility that other AGE compounds or tissue AGEs may contribute to disease outcomes. It has been shown that skin AGE levels (a marker of tissue AGE levels) measured by fluorescence are a predictor of mortality in haemodialysis patients [231]. As dietary AGEs contribute to total body levels of AGEs, restriction of intake provides an attractive and simple lifestyle modification that could reduce inflammation or improve outcomes in patients with CKD.

The majority of studies in animals support the role of dietary AGEs in the development and progression of CKD [232,233,234,235,236]. A high AGE diet was shown to increase proteinuria in healthy rats and 5/6 nephrectomised rats (an animal model of CKD) by 2-fold and 8-fold, respectively, suggesting that a high AGE intake may be more detrimental in cases of impaired renal function [233,234,236]. Supporting this evidence, another study reported that a high AGE diet accelerated the development of glomerulosclerosis, interstitial fibrosis and reduced creatinine clearance in 5/6 nephrectomised rats [232]. Results in mouse models of diabetic nephropathy have produced different results with one study showing a low AGE diet attenuated the development of albuminuria and glomerular sclerosis [235], while another found that AGE restriction had no effect on renal parameters, suggesting that diabetic and non-diabetic CKD may respond differently to dietary AGE intake [237].

There have only been two randomised parallel-arm interventions in humans that have compared the effect of a low AGE intake to high AGE intake in patients with CKD, both from the same research group [238,239]. In relation to the effect of dietary AGEs on progression of CKD, neither of these studies reported urinary albumin, or markers of glomerular filtration rate. Vlassara et al. measured creatinine clearance but, contrary to the evidence obtained from animal studies, reported no difference in changes in clearance after four weeks of a low AGE intake compared with a standard high AGE diet [239]. Uribarri et al. did, however, report reduced levels of CVD risk markers after consumption of a low AGE diet [238]. In a dietary trial in healthy obese men, a population at risk of developing CKD, it was observed that both the albumin-to-creatinine ratio and plasma cystatin C levels (a marker of GFR) were increased after two weeks’ consumption of a high AGE diet compared to a low AGE diet [240]. While these results appear to support the reduction in dietary AGE intake to improve some markers of renal function and risk of CVD, further research in this area is required before any recommendations can be made.

## 10. Conclusions

Diet remains an important factor in the prevention and management of CKD. Whilst there are well-established recommendations for protein intake in patients with CKD, emerging evidence indicates that the source of protein may be important, with a shift from animal to plant sources of protein seemingly beneficial. Furthermore, plant foods are higher in dietary fibre and there is accumulating evidence that increasing intake of dietary fibre and non-digestible carbohydrates improves biochemical markers in CKD patients; however, whether this translates to reductions in mortality levels has yet to be proven. Plant protein food sources also have lower organic phosphate bioavailability, providing yet another plausible mechanism by which plant protein appears to have greater benefit when compared to animal sources of protein. Inorganic phosphate consumption has continued to rise with increases in the consumption of preservative-rich convenience foods, and should be limited in the diet of the CKD patient.

Current recommendations for CKD patients include restricting sodium and potassium intake. Thus fruits and vegetables, sources of potassium, dietary fibre and plant protein have generally been somewhat restricted in CKD patients. There is increasing evidence to indicate that during early CKD a diet high in fruits and vegetables may delay progression of the disease; however, further studies should be completed before changes to recommendations occur. *n*-3 PUFAs, found in oily fish, reduce the risk of cardiovascular events and may reduce mortality in CKD patients. Vitamin D supplementation reduces proteinuria but does not translate into changes in cardiovascular or mortality outcomes. Excessive dietary fructose is a factor that may promote the progression of CKD; however, evidence is currently limited. For patients who have a high dietary fructose intake by way of consuming many sugar-sweetened beverages, there may be a benefit to decreasing intake. Dietary AGEs, formed in foods during high heat treatment such as baking, are prevalent in the Western diet and may contribute to the progression of CKD; however, further evidence is required to establish causation. Further high-quality studies are required to fully establish the role of these dietary factors in CKD patients.

## References

[1] Levey A.S., Coresh J. (2012). Chronic kidney disease. Lancet.

[2] Levey A.S., de Jong P.E., Coresh J., El Nahas M., Astor B.C., Matsushita K., Gansevoort R.T., Kasiske B.L., Eckardt K.-U. (2011). The definition, classification, and prognosis of chronic kidney disease: A KDIGO Controversies Conference report. Kidney Int..

[3] James M.T., Hemmelgarn B.R., Tonelli M. (2010). Early recognition and prevention of chronic kidney disease. Lancet.

[4] Weiner D.E., Tighiouart H., Amin M.G., Stark P.C., MacLeod B., Griffith J.L., Salem D.N., Levey A.S., Sarnak M.J. (2004). Chronic kidney disease as a risk factor for cardiovascular disease and all-cause mortality: A pooled analysis of community-based studies. J. Am. Soc. Nephrol..

[5] Eckardt K.-U., Coresh J., Devuyst O., Johnson R.J., Köttgen A., Levey A.S., Levin A. (2013). Evolving importance of kidney disease: From subspecialty to global health burden. Lancet.

[6] Cordain L., Eaton S.B., Sebastian A., Mann N., Lindeberg S., Watkins B.A., O’Keefe J.H., Brand-Miller J. (2005). Origins and evolution of the Western diet: Health implications for the 21st century. Am. J. Clin. Nutr..

[7] Odermatt A. (2011). The Western-style diet: A major risk factor for impaired kidney function and chronic kidney disease. Am. J. Physiol. Ren. Physiol..

[8] (2013). Kidney Disease: Improving Global Outcomes (KDIGO) CKD Work Group: KDIGO clinical practice guideline for the evaluation and management of chronic kidney disease. Kidney Int. Suppl..

[9] Dunkler D., Kohl M., Teo K.K., Heinze G., Dehghan M., Clase C.M., Gao P., Yusuf S., Mann J.F., Oberbauer R. (2015). Dietary risk factors for incidence or progression of chronic kidney disease in individuals with type 2 diabetes in the European Union. Nephrol. Dial. Transplant..

[10] Diamond J.R. (1990). Effects of dietary interventions on glomerular pathophysiology. Am. J. Physiol. Ren. Physiol..

[11] Fouque D., Aparicio M. (2007). Eleven reasons to control the protein intake of patients with chronic kidney disease. Nat. Clin. Pract. Nephrol..

[12] Kaysen G.A., Gambertoglio J., Jimenez I., Jones H., Hutchison F.N. (1986). Effect of dietary protein intake on albumin homeostasis in nephrotic patients. Kidney Int..

[13] Edwards C.A., Parrett A., Khanna S. (2006). Nondigestible Carbohydrates. Carbohydrates in Food.

[14] Ash S., Campbell K.L., Bogard J., Millichamp A. (2014). Nutrition prescription to achieve positive outcomes in chronic kidney disease: A systematic review. Nutrients.

[15] Bellizzi V., Di Iorio B.R., De Nicola L., Minutolo R., Zamboli P., Trucillo P., Catapano F., Cristofano C., Scalfi L., Conte G. (2007). Very low protein diet supplemented with ketoanalogs improves blood pressure control in chronic kidney disease. Kidney Int..

[16] Di Iorio B.R., Bellizzi V., Bellasi A., Torraca S., D’Arrigo G., Tripepi G., Zoccali C. (2013). Phosphate attenuates the anti-proteinuric effect of very low-protein diet in CKD patients. Nephrol. Dial. Transplant..

[17] Schwingshackl L., Hoffmann G. (2014). Comparison of high vs. normal/low protein diets on renal function in subjects without chronic kidney disease: A systematic review and meta-analysis. PLoS ONE.

[18] Jarusiripipat C., Shapiro J.I., Chan L., Schrier R.W. (1991). Reduction of remnant nephron hypermetabolism by protein restriction. Am. J. Kidney Dis..

[19] Kleinknecht C., Salusky I., Broyer M., Gubler M.-C. (1979). Effect of various protein diets on growth, renal function, and survival of uremic rats. Kidney Int..

[20] Kenner C.H., Evan A.P., Blomgren P., Aronoff G.R., Luft F.C. (1985). Effect of protein intake on renal function and structure in partially nephrectomized rats. Kidney Int..

[21] Hostetter T.H., Meyer T.W., Rennke H.G., Brenner B.M., Noddin J.A., Sandstrom D.J. (1986). Chronic effects of dietary protein in the rat with intact and reduced renal mass. Kidney Int..

[22] Robertson L.M., Waugh N., Robertson A. (2007). Protein restriction for diabetic renal disease. Cochrane Libr..

[23] Williams P., Stevens M., Fass G., Irons L., Bone J. (1991). Failure of dietary protein and phosphate restriction to retard the rate of progression of chronic renal failure: A prospective, randomized, controlled trial. QJM.

[24] Klahr S., Levey A.S., Beck G.J., Caggiula A.W., Hunsicker L., Kusek J.W., Striker G. (1994). The effects of dietary protein restriction and blood-pressure control on the progression of chronic renal disease. N. Engl. J. Med..

[25] Carrero J.J., Stenvinkel P., Cuppari L., Ikizler T.A., Kalantar-Zadeh K., Kaysen G., Mitch W.E., Price S.R., Wanner C., Wang A.Y.M. (2013). Etiology of the Protein-Energy Wasting Syndrome in Chronic Kidney Disease: A Consensus Statement From the International Society of Renal Nutrition and Metabolism (ISRNM). J. Ren. Nutr..

[26] Menon V., Kopple J.D., Wang X., Beck G.J., Collins A.J., Kusek J.W., Greene T., Levey A.S., Sarnak M.J. (2009). Effect of a very low-protein diet on outcomes: Long-term follow-up of the Modification of Diet in Renal Disease (MDRD) Study. Am. J. Kidney Dis..

[27] Dobre M., Yang W., Chen J., Drawz P., Hamm L.L., Horwitz E., Hostetter T., Jaar B., Lora C.M., Nessel L. (2013). Association of Serum Bicarbonate With Risk of Renal and Cardiovascular Outcomes in CKD: A Report From the Chronic Renal Insufficiency Cohort (CRIC) Study. Am. J. Kidney Dis..

[28] Shah S.N., Abramowitz M., Hostetter T.H., Melamed M.L. (2009). Serum Bicarbonate Levels and the Progression of Kidney Disease: A Cohort Study. Am. J. Kidney Dis..

[29] Kovesdy C.P., Anderson J.E., Kalantar-Zadeh K. (2009). Association of serum bicarbonate levels with mortality in patients with non-dialysis-dependent CKD. Nephrol. Dial. Transplant..

[30] Adeva M.M., Souto G. (2011). Diet-induced metabolic acidosis. Clin. Nutr..

[31] Alpern R.J., Sakhaee K. (1997). The clinical spectrum of chronic metabolic acidosis: Homeostatic mechanisms produce significant morbidity. Am. J. Kidney Dis..

[32] Kurtz I. (1991). Role of Ammonia in the Induction of Renal Hypertrophy. Am. J. Kidney Dis..

[33] Mitch W.E., Medina R., Grieber S., May R.C., England B.K., Price S.R., Bailey J.L., Goldberg A.L. (1994). Metabolic acidosis stimulates muscle protein degradation by activating the adenosine triphosphate-dependent pathway involving ubiquitin and proteasomes. J. Clin. Investig..

[34] Scialla J.J., Appel L.J., Wolf M., Yang W., Zhang X., Sozio S.M., Miller E.R., Bazzano L.A., Cuevas M., Glenn M.J. (2012). Plant Protein Intake Is Associated with Fibroblast Growth Factor 23 and Serum Bicarbonate in Patients with CKD: The Chronic Renal Insufficiency Cohort Study. J. Ren. Nutr..

[35] Azadbakht L., Atabak S., Esmaillzadeh A. (2008). Soy Protein Intake, Cardiorenal Indices, and C-Reactive Protein in Type 2 Diabetes With Nephropathy: A longitudinal randomized clinical trial. Diabetes Care.

[36] Kontessis P., Jones S., Dodds R., Trevisan R., Nosadini R., Fioretto P., Borsato M., Sacerdoti D., Viberti G. (1990). Renal, metabolic and hormonal responses to ingestion of animal and vegetable proteins. Kidney Int..

[37] Garneata L., Stancu A., Dragomir D., Stefan G., Mircescu G. (2016). Ketoanalogue-Supplemented Vegetarian Very Low–Protein Diet and CKD Progression. J. Am. Soc. Nephrol..

[38] Di Iorio B., Di Micco L., Marzocco S., De Simone E., De Blasio A., Sirico M., Nardone L. (2017). Very Low-Protein Diet (VLPD) Reduces Metabolic Acidosis in Subjects with Chronic Kidney Disease: The “Nutritional Light Signal” of the Renal Acid Load. Nutrients.

[39] Goraya N., Simoni J., Jo C.-H., Wesson D.E. (2014). Treatment of metabolic acidosis in patients with stage 3 chronic kidney disease with fruits and vegetables or oral bicarbonate reduces urine angiotensinogen and preserves glomerular filtration rate. Kidney Int..

[40] Goraya N., Simoni J., Jo C.-H., Wesson D.E. (2013). A Comparison of Treating Metabolic Acidosis in CKD Stage 4 Hypertensive Kidney Disease with Fruits and Vegetables or Sodium Bicarbonate. Clin. J. Am. Soc. Nephrol..

[41] Meijers B.K., Evenepoel P. (2011). The gut–kidney axis: Indoxyl sulfate, p-cresyl sulfate and CKD progression. Nephrol. Dial. Transplant..

[42] Niwa T. (2010). Indoxyl sulfate is a nephro-vascular toxin. J. Ren. Nutr..

[43] Tumur Z., Shimizu H., Enomoto A., Miyazaki H., Niwa T. (2010). Indoxyl sulfate upregulates expression of ICAM-1 and MCP-1 by oxidative stress-induced NF-ĸB activation. Am. J. Nephrol..

[44] Bammens B., Evenepoel P., Keuleers H., Verbeke K., Vanrenterghem Y. (2006). Free serum concentrations of the protein-bound retention solute p-cresol predict mortality in hemodialysis patients. Kidney Int..

[45] Liabeuf S., Barreto D.V., Barreto F.C., Meert N., Glorieux G., Schepers E., Temmar M., Choukroun G., Vanholder R., Massy Z.A. (2010). Free p-cresylsulphate is a predictor of mortality in patients at different stages of chronic kidney disease. Nephrol. Dial. Transplant..

[46] Patel K.P., Luo F.J.-G., Plummer N.S., Hostetter T.H., Meyer T.W. (2012). The Production of p-Cresol Sulfate and Indoxyl Sulfate in Vegetarians versus Omnivores. Clin. J. Am. Soc. Nephrol..

[47] Kandouz S., Mohamed A.S., Zheng Y., Sandeman S., Davenport A. (2016). Reduced protein bound uraemic toxins in vegetarian kidney failure patients treated by haemodiafiltration. Hemodial. Int..

[48] Koeth R.A., Wang Z., Levison B.S., Buffa J.A., Org E., Sheehy B.T., Britt E.B., Fu X., Wu Y., Li L. (2013). Intestinal microbiota metabolism of L-carnitine, a nutrient in red meat, promotes atherosclerosis. Nat. Med..

[49] Rampton D.S., Cohen S.L., Crammond V.D., Gibbons J., Lilburn M.F., Rabet J.Y., Vince A.J., Wager J.D., Wrong O.M. (1984). Treatment of chronic renal failure with dietary fiber. Clin. Nephrol..

[50] Vasilis F., Dimosthenis V. (2009). Inflammatory Syndrome in Chronic Kidney Disease: Pathogenesis and Influence on Outcomes. Inflamm. Allergy-Drug Targets.

[51] Krishnamurthy V.M.R., Wei G., Baird B.C., Murtaugh M., Chonchol M.B., Raphael K.L., Greene T., Beddhu S. (2012). High dietary fiber intake is associated with decreased inflammation and all-cause mortality in patients with chronic kidney disease. Kidney Int..

[52] Evenepoel P., Meijers B.K. (2012). Dietary fiber and protein: Nutritional therapy in chronic kidney disease and beyond. Kidney Int..

[53] Salmean Y.A., Segal M.S., Langkamp-Henken B., Canales M.T., Zello G.A., Dahl W.J. (2013). Foods With Added Fiber Lower Serum Creatinine Levels in Patients With Chronic Kidney Disease. J. Ren. Nutr..

[54] Salmean Y.A., Segal M.S., Palii S.P., Dahl W.J. (2015). Fiber Supplementation Lowers Plasma p-Cresol in Chronic Kidney Disease Patients. J. Ren. Nutr..

[55] Bliss D.Z., Stein T.P., Schleifer C.R., Settle R.G. (1996). Supplementation with gum arabic fiber increases fecal nitrogen excretion and lowers serum urea nitrogen concentration in chronic renal failure patients consuming a low-protein diet. Am. J. Clin. Nutr..

[56] Ali A.A., Ali K.E., Fadlalla A.E., Khalid K.E. (2007). The effects of gum arabic oral treatment on the metabolic profile of chronic renal failure patients under regular haemodialysis in Central Sudan. Nat. Prod. Res..

[57] Chiavaroli L., Mirrahimi A., Sievenpiper J.L., Jenkins D.J.A., Darling P.B. (2015). Dietary fiber effects in chronic kidney disease: A systematic review and meta-analysis of controlled feeding trials. Eur. J. Clin. Nutr..

[58] Meijers B.K.I., De Preter V., Verbeke K., Vanrenterghem Y., Evenepoel P. (2010). p-Cresyl sulfate serum concentrations in haemodialysis patients are reduced by the prebiotic oligofructose-enriched inulin. Nephrol. Dial. Transplant..

[59] Nakabayashi I., Nakamura M., Kawakami K., Ohta T., Kato I., Uchida K., Yoshida M. (2011). Effects of synbiotic treatment on serum level of p-cresol in haemodialysis patients: A preliminary study. Nephrol. Dial. Transplant..

[60] Sirich T.L., Plummer N.S., Gardner C.D., Hostetter T.H., Meyer T.W. (2014). Effect of Increasing Dietary Fiber on Plasma Levels of Colon-Derived Solutes in Hemodialysis Patients. Clin. J. Am. Soc. Nephrol..

[61] Johnson D.W., Atai E., Chan M., Phoon R.K.S., Scott C., Toussaint N.D., Turner G.L., Usherwood T., Wiggins K.J. (2013). KHA-CARI Guideline: Early chronic kidney disease: Detection, prevention and management. Nephrology.

[62] Cupisti A., D’Alessandro C., Valeri A., Capitanini A., Meola M., Betti G., Barsotti G. (2010). Food Intake and Nutritional Status in Stable Hemodialysis Patients. Ren. Fail..

[63] Kalantar-Zadeh K., Kopple J.D., Deepak S., Block D., Block G. (2002). Food intake characteristics of hemodialysis patients as obtained by food frequency questionnaire. J. Ren. Nutr..

[64] Khoueiry G., Waked A., Goldman M., El-Charabaty E., Dunne E., Smith M., Kleiner M., Lafferty J., Kalantar-Zadeh K., El-Sayegh S. (2011). Dietary Intake in Hemodialysis Patients Does Not Reflect a Heart Healthy Diet. J. Ren. Nutr..

[65] Rossi M., Klein K., Johnson D.W., Campbell K.L. (2012). Pre-, Pro-, and Synbiotics: Do They Have a Role in Reducing Uremic Toxins? A Systematic Review and Meta-Analysis. Int. J. Nephrol..

[66] Salmean Y.A., Zello G.A., Dahl W.J. (2013). Foods with added fiber improve stool frequency in individuals with chronic kidney disease with no impact on appetite or overall quality of life. BMC Res. Notes.

[67] Grabitske H.A., Slavin J.L. (2009). Gastrointestinal Effects of Low-Digestible Carbohydrates. Crit. Rev. Food Sci. Nutr..

[68] Gansevoort R.T., Correa-Rotter R., Hemmelgarn B.R., Jafar T.H., Heerspink H.J.L., Mann J.F., Matsushita K., Wen C.P. (2013). Chronic kidney disease and cardiovascular risk: Epidemiology, mechanisms, and prevention. Lancet.

[69] Hultström M. (2012). Development of structural kidney damage in spontaneously hypertensive rats. J. Hypertens..

[70] Brenner B.M., Garcia D.L., Anderson S. (1988). Glomeruli and blood pressure Less of one, more the other?. Am. J. Hypertens..

[71] Jones-Burton C., Mishra S.I., Fink J.C., Brown J., Gossa W., Bakris G.L., Weir M.R. (2006). An in-depth review of the evidence linking dietary salt intake and progression of chronic kidney disease. Am. J. Nephrol..

[72] McMahon E.J., Bauer J.D., Hawley C.M., Isbel N.M., Stowasser M., Johnson D.W., Campbell K.L. (2013). A randomized trial of dietary sodium restriction in CKD. J. Am. Soc. Nephrol..

[73] Slagman M.C.J., Waanders F., Hemmelder M.H., Woittiez A.-J., Janssen W.M.T., Lambers Heerspink H.J., Navis G., Laverman G.D. (2011). Moderate dietary sodium restriction added to angiotensin converting enzyme inhibition compared with dual blockade in lowering proteinuria and blood pressure: Randomised controlled trial. BMJ.

[74] Adrogué H.J., Madias N.E. (2007). Sodium and Potassium in the Pathogenesis of Hypertension. N. Engl. J. Med..

[75] Tyson C.C., Kuchibhatla M., Patel U.D., Pun P.H., Chang A., Nwankwo C., Joseph M.A., Svetkey L.P. (2014). Impact of Kidney Function on Effects of the Dietary Approaches to Stop Hypertension (Dash) Diet. J. Hypertens..

[76] Noori N., Kalantar-Zadeh K., Kovesdy C.P., Murali S.B., Bross R., Nissenson A.R., Kopple J.D. (2010). Dietary potassium intake and mortality in long-term hemodialysis patients. Am. J. Kidney Dis..

[77] Cheng J., Zhang W., Zhang X., Li X., Chen J. (2012). Efficacy and Safety of Paricalcitol Therapy for Chronic Kidney Disease: A Meta-Analysis. Clin. J. Am. Soc. Nephrol..

[78] Tomasello S.P.B. (2008). Secondary Hyperparathyroidism and Chronic Kidney Disease. Diabetes Spectr..

[79] Saravanan P., Davidson N.C. (2010). Risk Assessment for Sudden Cardiac Death in Dialysis Patients. Circ. Arrhythm. Electrophysiol..

[80] Drechsler C., Pilz S., Obermayer-Pietsch B., Verduijn M., Tomaschitz A., Krane V., Espe K., Dekker F., Brandenburg V., März W. (2010). Vitamin D deficiency is associated with sudden cardiac death, combined cardiovascular events, and mortality in haemodialysis patients. Eur. Heart J..

[81] Pilz S., Iodice S., Zittermann A., Grant W.B., Gandini S. (2011). Vitamin D Status and Mortality Risk in CKD: A Meta-analysis of Prospective Studies. Am. J. Kidney Dis..

[82] Goodman W.G., Goldin J., Kuizon B.D., Yoon C., Gales B., Sider D., Wang Y., Chung J., Emerick A., Greaser L. (2000). Coronary-Artery Calcification in Young Adults with End-Stage Renal Disease Who Are Undergoing Dialysis. N. Engl. J. Med..

[83] Block G.A., Hulbert-Shearon T.E., Levin N.W., Port F.K. (1998). Association of serum phosphorus and calcium x phosphate product with mortality risk in chronic hemodialysis patients: A national study. Am. J. Kidney Dis..

[84] Keyzer C.A., Lambers-Heerspink H.J., Joosten M.M., Deetman P.E., Gansevoort R.T., Navis G., Kema I.P., de Zeeuw D., Bakker S.J., de Borst M.H. (2015). Plasma Vitamin D Level and Change in Albuminuria and eGFR According to Sodium Intake. Clin. J. Am. Soc. Nephrol..

[85] Xu L., Wan X., Huang Z., Zeng F., Wei G., Fang D., Deng W., Li Y. (2013). Impact of Vitamin D on Chronic Kidney Diseases in Non-Dialysis Patients: A Meta-Analysis of Randomized Controlled Trials. PLoS ONE.

[86] de Boer I.H., Sachs M., Hoofnagle A.N., Utzschneider K.M., Kahn S.E., Kestenbaum B., Himmelfarb J. (2013). Paricalcitol does not improve glucose metabolism in patients with stage 3–4 chronic kidney disease. Kidney Int..

[87] Fishbane S., Chittineni H., Packman M., Dutka P., Ali N., Durie N. (2009). Oral Paricalcitol in the Treatment of Patients With CKD and Proteinuria: A Randomized Trial. Am. J. Kidney Dis..

[88] de Zeeuw D., Agarwal R., Amdahl M., Audhya P., Coyne D., Garimella T., Parving H.-H., Pritchett Y., Remuzzi G., Ritz E. (2010). Selective vitamin D receptor activation with paricalcitol for reduction of albuminuria in patients with type 2 diabetes (VITAL study): A randomised controlled trial. Lancet.

[89] Alborzi P., Patel N.A., Peterson C., Bills J.E., Bekele D.M., Bunaye Z., Light R.P., Agarwal R. (2008). Paricalcitol Reduces Albuminuria and Inflammation in Chronic Kidney Disease: A Randomized Double-Blind Pilot Trial. Hypertension.

[90] Kandula P., Dobre M., Schold J.D., Schreiber M.J., Mehrotra R., Navaneethan S.D. (2011). Vitamin D Supplementation in Chronic Kidney Disease: A Systematic Review and Meta-Analysis of Observational Studies and Randomized Controlled Trials. Clin. J. Am. Soc. Nephrol..

[91] Han T., Rong G., Quan D., Shu Y., Liang Z., She N., Liu M., Yang B., Cheng G., Lv Y. (2013). Meta-Analysis: The Efficacy and Safety of Paricalcitol for the Treatment of Secondary Hyperparathyroidism and Proteinuria in Chronic Kidney Disease. BioMed Res. Int..

[92] de Zeeuw D., Remuzzi G., Parving H.-H., Keane W.F., Zhang Z., Shahinfar S., Snapinn S., Cooper M.E., Mitch W.E., Brenner B.M. (2004). Proteinuria, a target for renoprotection in patients with type 2 diabetic nephropathy: Lessons from RENAAL. Kidney Int..

[93] Agarwal R., Acharya M., Tian J., Hippensteel R.L., Melnick J.Z., Qiu P., Williams L., Batlle D. (2005). Antiproteinuric effect of oral paricalcitol in chronic kidney disease. Kidney Int..

[94] de Borst M.H., Hajhosseiny R., Tamez H., Wenger J., Thadhani R., Goldsmith D.J.A. (2013). Active Vitamin D Treatment for Reduction of Residual Proteinuria: A Systematic Review. J. Am. Soc. Nephrol..

[95] Ruggenenti P., Perna A., Gherardi G., Garini G., Zoccali C., Salvadori M., Scolari F., Schena F.P., Remuzzi G. (1999). Renoprotective properties of ACE-inhibition in non-diabetic nephropathies with non-nephrotic proteinuria. Lancet.

[96] Brenner B.M., Cooper M.E., de Zeeuw D., Keane W.F., Mitch W.E., Parving H.-H., Remuzzi G., Snapinn S.M., Zhang Z., Shahinfar S. (2001). Effects of Losartan on Renal and Cardiovascular Outcomes in Patients with Type 2 Diabetes and Nephropathy. N. Engl. J. Med..

[97] Zheng Z., Shi H., Jia J., Li D., Lin S. (2013). Vitamin D supplementation and mortality risk in chronic kidney disease: A meta-analysis of 20 observational studies. BMC Nephrol..

[98] Mann M.C., Hobbs A.J., Hemmelgarn B.R., Roberts D.J., Ahmed S.B., Rabi D.M. (2015). Effect of oral vitamin D analogus on mortality and cardiovascular outcomes among adults with chronic kidney disease: A meta-analysis. Clin. Kidney J..

[99] Morrone L.F., Cozzolino M. (2015). The beneficial impact of vitamin D treatment in CKD patients: What’s next?. Clin. Kidney J..

[100] Theodoratou E., Tzoulaki I., Zgaga L., Ioannidis J.P.A. (2014). Vitamin D and multiple health outcomes: Umbrella review of systematic reviews and meta-analyses of observational studies and randomised trials. BMJ.

[101] Blaine J., Chonchol M., Levi M. (2015). Renal Control of Calcium, Phosphate, and Magnesium Homeostasis. Clin. J. Am. Soc. Nephrol..

[102] Bover J., Andrés E., Lloret M.J., Aguilar A., Ballarín J. (2009). Dietary and Pharmacological Control of Calcium and Phosphate Metabolism in Dialysis Patients. Blood Purif..

[103] Levin A., Bakris G.L., Molitch M., Smulders M., Tian J., Williams L.A., Andress D.L. (2006). Prevalence of abnormal serum vitamin D, PTH, calcium, and phosphorus in patients with chronic kidney disease: Results of the study to evaluate early kidney disease. Kidney Int..

[104] Achinger S.G., Ayus J.C. (2006). Left Ventricular Hypertrophy: Is Hyperphosphatemia among Dialysis Patients a Risk Factor?. J. Am. Soc. Nephrol..

[105] Hruska K.A., Saab G., Mathew S., Lund R. (2007). Phosphorus Metabolism and Management in Chronic Kidney Disease: Renal Osteodystrophy, Phosphate Homeostasis, and Vascular Calcification. Semin. Dial..

[106] Voormolen N., Noordzij M., Grootendorst D.C., Beetz I., Sijpkens Y.W., van Manen J.G., Boeschoten E.W., Huisman R.M., Krediet R.T., Dekker F.W. (2007). High plasma phosphate as a risk factor for decline in renal function and mortality in pre-dialysis patients. Nephrol. Dial. Transplant..

[107] Kestenbaum B., Sampson J.N., Rudser K.D., Patterson D.J., Seliger S.L., Young B., Sherrard D.J., Andress D.L. (2005). Serum Phosphate Levels and Mortality Risk among People with Chronic Kidney Disease. J. Am. Soc. Nephrol..

[108] Eddington H., Hoefield R., Sinha S., Chrysochou C., Lane B., Foley R.N., Hegarty J., New J., O’Donoghue D.J., Middleton R.J. (2010). Serum Phosphate and Mortality in Patients with Chronic Kidney Disease. Clin. J. Am. Soc. Nephrol..

[109] Block G.A., Klassen P.S., Lazarus J.M., Ofsthun N., Lowrie E.G., Chertow G.M. (2004). Mineral Metabolism, Mortality, and Morbidity in Maintenance Hemodialysis. J. Am. Soc. Nephrol..

[110] Young E.W., Albert J.M., Satayathum S., Goodkin D.A., Pisoni R.L., Akiba T., Akizawa T., Kurokawa K., Bommer J., Piera L. (2005). Predictors and consequences of altered mineral metabolism: The Dialysis Outcomes and Practice Patterns Study. Kidney Int..

[111] Ganesh S.K., Stack A.G., Levin N.W., Hulbert-Shearon T.E., Port F.K. (2001). Association of Elevated Serum PO4, Ca × PO4 Product, and Parathyroid Hormone with Cardiac Mortality Risk in Chronic Hemodialysis Patients. J. Am. Soc. Nephrol..

[112] Kovesdy C., Kalantar-Zadeh K. (2008). Bone and mineral disorders in pre-dialysis CKD. Int. Urol. Nephrol..

[113] Schwarz S., Trivedi B.K., Kalantar-Zadeh K., Kovesdy C.P. (2006). Association of Disorders in Mineral Metabolism with Progression of Chronic Kidney Disease. Clin. J. Am. Soc. Nephrol..

[114] Zoccali C., Ruggenenti P., Perna A., Leonardis D., Tripepi R., Tripepi G., Mallamaci F., Remuzzi G. (2011). Phosphate May Promote CKD Progression and Attenuate Renoprotective Effect of ACE Inhibition. J. Am. Soc. Nephrol..

[115] Chue C.D., Edwards N.C., Davis L.J., Steeds R.P., Townend J.N., Ferro C.J. (2011). Serum phosphate but not pulse wave velocity predicts decline in renal function in patients with early chronic kidney disease. Nephrol. Dial. Transplant..

[116] Sim J.J., Bhandari S.K., Smith N., Chung J., Liu I.L.A., Jacobsen S.J., Kalantar-Zadeh K. (2013). Phosphorus and Risk of Renal Failure in Subjects with Normal Renal Function. Am. J. Med..

[117] Kalantar-Zadeh K., Gutekunst L., Mehrotra R., Kovesdy C.P., Bross R., Shinaberger C.S., Noori N., Hirschberg R., Benner D., Nissenson A.R. (2010). Understanding Sources of Dietary Phosphorus in the Treatment of Patients with Chronic Kidney Disease. Clin. J. Am. Soc. Nephrol..

[118] Kloppenburg W.D., Stegeman C.A., Kremer Hovinga T.K., Vastenburg G., Vos P., de Jong P.E., Huisman R.M. (2004). Effect of prescribing a high protein diet and increasing the dose of dialysis on nutrition in stable chronic haemodialysis patients: A randomized, controlled trial. Nephrol. Dial. Transplant..

[119] Cupisti A., Kalantar-Zadeh K. (2013). Management of Natural and Added Dietary Phosphorus Burden in Kidney Disease. Semin. Nephrol..

[120] Uribarri J., Calvo M.S. (2003). Hidden Sources of Phosphorus in the Typical American Diet: Does it Matter in Nephrology?. Semin. Dial..

[121] Calvo M.S., Moshfegh A.J., Tucker K.L. (2014). Assessing the Health Impact of Phosphorus in the Food Supply: Issues and Considerations. Adv. Nutr. Int. Rev. J..

[122] Sinha A., Prasad N. (2014). Dietary management of hyperphosphatemia in chronic kidney disease. Clin. Queries Nephrol..

[123] Nadkarni G.N., Uribarri J. (2014). Phosphorus and the Kidney: What Is Known and What Is Needed. Adv. Nutr. Int. Rev. J..

[124] Takeda E., Yamamoto H., Yamanaka-Okumura H., Taketani Y. (2014). Increasing Dietary Phosphorus Intake from Food Additives: Potential for Negative Impact on Bone Health. Adv. Nutr. Int. Rev. J..

[125] McCutcheon J., Campbell K., Ferguson M., Day S., Rossi M. (2015). Prevalence of Phosphorus-Based Additives in the Australian Food Supply: A Challenge for Dietary Education?. J. Ren. Nutr..

[126] Guida B., Piccoli A., Trio R., Laccetti R., Nastasi A., Paglione A., Memoli A., Memoli B. (2011). Dietary phosphate restriction in dialysis patients: A new approach for the treatment of hyperphosphataemia. Nutr. Metab. Cardiovasc. Dis..

[127] Sullivan C., Sayre S.S., Leon J.B., Machekano R., Love T.E., Porter D., Marbury M., Sehgal A.R. (2009). Effect of food additives on hyperphosphatemia among patients with end-stage renal disease: A randomized controlled trial. JAMA.

[128] Shinaberger C.S., Greenland S., Kopple J.D., Van Wyck D., Mehrotra R., Kovesdy C.P., Kalantar-Zadeh K. (2008). Is controlling phosphorus by decreasing dietary protein intake beneficial or harmful in persons with chronic kidney disease?. Am. J. Clin. Nutr..

[129] Lynch K.E., Lynch R., Curhan G.C., Brunelli S.M. (2011). Prescribed Dietary Phosphate Restriction and Survival among Hemodialysis Patients. Clin. J. Am. Soc. Nephrol..

[130] Sherman R.A. (2007). Dietary Phosphate Restriction and Protein Intake in Dialysis Patients: A Misdirected Focus. Semin. Dial..

[131] Benini O., D’Alessandro C., Gianfaldoni D., Cupisti A. (2011). Extra-Phosphate Load from Food Additives in Commonly Eaten Foods: A Real and Insidious Danger for Renal Patients. J. Ren. Nutr..

[132] Sherman R.A., Mehta O. (2009). Phosphorus and Potassium Content of Enhanced Meat and Poultry Products: Implications for Patients Who Receive Dialysis. Clin. J. Am. Soc. Nephrol..

[133] Zhao L.G., Sun J.W., Yang Y., Ma X., Wang Y.Y., Xiang Y.B. (2016). Fish consumption and all-cause mortality: A meta-analysis of cohort studies. Eur. J. Clin. Nutr..

[134] Shapiro H., Theilla M., Attal-Singer J., Singer P. (2011). Effects of polyunsaturated fatty acid consumption in diabetic nephropathy. Nat. Rev. Nephrol..

[135] Fassett R.G., Gobe G.C., Peake J.M., Coombes J.S. (2010). Omega-3 Polyunsaturated Fatty Acids in the Treatment of Kidney Disease. Am. J. Kidney Dis..

[136] Christensen J.H., Schmidt E.B., Svensson M. (2011). *n*-3 polyunsaturated fatty acids, lipids and lipoproteins in end-stage renal disease. Clin. Lipidol..

[137] Eslick G.D., Howe P.R.C., Smith C., Priest R., Bensoussan A. (2009). Benefits of fish oil supplementation in hyperlipidemia: A systematic review and meta-analysis. Int. J. Cardiol..

[138] Mori T.A., Burke V., Puddey I., Irish A., Cowpland C.A., Beilin L., Dogra G., Watts G.F. (2009). The effects of [omega]3 fatty acids and coenzyme Q10 on blood pressure and heart rate in chronic kidney disease: A randomized controlled trial. J. Hypertens..

[139] Svensson M., Christensen J.H., Sølling J., Schmidt E.B. (2004). The effect of *n*-3 fatty acids on plasma lipids and lipoproteins and blood pressure in patients with CRF. Am. J. Kidney Dis..

[140] Guebre-Egziabher F., Debard C., Drai J., Denis L., Pesenti S., Bienvenu J., Vidal H., Laville M., Fouque D. (2013). Differential dose effect of fish oil on inflammation and adipose tissue gene expression in chronic kidney disease patients. Nutrition.

[141] Svensson M., Schmidt E.B., Jørgensen K.A., Christensen J.H. (2008). The effect of *n*-3 fatty acids on lipids and lipoproteins in patients treated with chronic haemodialysis: A randomized placebo-controlled intervention study. Nephrol. Dial. Transplant..

[142] Khajehdehi P. (2000). Lipid-lowering effect of polyunsaturated fatty acids in hemodialysis patients. J. Ren. Nutr..

[143] Ando M., Sanaka T., Nihei H. (1999). Eicosapentanoic Acid Reduces Plasma Levels of Remnant Lipoproteins and Prevents in Vivo Peroxidation of LDL in Dialysis Patients. J. Am. Soc. Nephrol..

[144] Kooshki A., Taleban F.A., Tabibi H., Hedayati M. (2011). Effects of Omega-3 Fatty Acids on Serum Lipids, Lipoprotein (a), and Hematologic Factors in Hemodialysis Patients. Ren. Fail..

[145] Saifullah A., Watkins B.A., Saha C., Li Y., Moe S.M., Friedman A.N. (2007). Oral fish oil supplementation raises blood omega-3 levels and lowers C-reactive protein in haemodialysis patients—A pilot study. Nephrol. Dial. Transplant..

[146] Perunicic-Pekovic G.B., Rasic Z.R., Pljesa S.I., Sobajic S.S., Djuricic I., Maletic R., Ristic-Medic D.K. (2007). Effect of *n*-3 fatty acids on nutritional status and inflammatory markers in haemodialysis patients. Nephrology.

[147] Taziki O., Lessan-Pezeshki M., Akha O., Vasheghani F. (2007). The Effect of Low Dose Omega-3 on Plasma Lipids in Hemodialysis Patients. Saudi J. Kidney Dis. Transpl..

[148] Poulia K.-A., Panagiotakos D.B., Tourlede E., Rezou A., Stamatiadis D., Boletis J., Zampelas A. (2011). Omega-3 Fatty Acids Supplementation Does Not Affect Serum Lipids in Chronic Hemodialysis Patients. J. Ren. Nutr..

[149] Donnelly S.M., Ali M.A., Churchill D.N. (1992). Effect of *n*-3 fatty acids from fish oil on hemostasis, blood pressure, and lipid profile of dialysis patients. J. Am. Soc. Nephrol..

[150] Schmitz P.G., McCloud L.K., Reikes S.T., Leonard C.L., Gellens M.E. (2002). Prophylaxis of Hemodialysis Graft Thrombosis with Fish Oil: Double-Blind, Randomized, Prospective Trial. J. Am. Soc. Nephrol..

[151] Bowden R.G., Jitomir J., Wilson R.L., Gentile M. (2009). Effects of Omega-3 Fatty Acid Supplementation on Lipid Levels in Endstage Renal Disease Patients. J. Ren. Nutr..

[152] Tayebi-Khosroshahi H., Dehgan R., Habibi Asl B., Safaian A., Panahi F., Estakhri R., Purasgar B. (2013). Effect of omega-3 supplementation on serum level of homocysteine in hemodialysis patients. Iran J. Kidney Dis..

[153] Rasic-Milutinovic Z., Perunicic G., Pljesa S., Gluvic Z., Sobajic S., Djuric I., Ristic D. (2007). Effects of *N*-3 PUFAs Supplementation on Insulin Resistance and Inflammatory Biomarkers in Hemodialysis Patients. Ren. Fail..

[154] An W.S., Lee S.M., Son Y.K., Kim S.E., Kim K.H., Han J.Y., Bae H.R., Rha S.H., Park Y. (2012). Omega-3 fatty acid supplementation increases 1,25-dihydroxyvitamin D and fetuin-A levels in dialysis patients. Nutr. Res..

[155] Bowden R.G., Wilson R.L., Deike E., Gentile M. (2009). Fish Oil Supplementation Lowers C-Reactive Protein Levels Independent of Triglyceride Reduction in Patients With End-Stage Renal Disease. Nutr. Clin. Pract..

[156] Lok C.E., Moist L., Hemmelgarn B.R., Tonelli M., Vazquez M.A., Dorval M., Oliver M., Donnelly S., Allon M., Stanley K. (2012). Effect of fish oil supplementation on graft patency and cardiovascular events among patients with new synthetic arteriovenous hemodialysis grafts: A randomized trial. JAMA.

[157] Bessell E., Jose M.D., McKercher C. (2015). Associations of fish oil and vitamin B and E supplementation with cardiovascular outcomes and mortality in people receiving haemodialysis: A review. BMC Nephrol..

[158] Zhu W., Dong C., Du H., Zhang H., Chen J., Hu X., Hu F. (2014). Effects of fish oil on serum lipid profile in dialysis patients: A systematic review and meta-analysis of randomized controlled trials. Lipids Health Dis..

[159] Schuchardt J.P., Hahn A. (2013). Bioavailability of long-chain omega-3 fatty acids. Prostaglandins Leukot. Essent. Fat. Acids.

[160] Khawaja O.A., Gaziano J.M., Djoussé L. (2014). *N*-3 Fatty Acids for Prevention of Cardiovascular Disease. Curr. Atheroscler. Rep..

[161] Chi H., Lin X., Huang H., Zheng X., Li T., Zou Y. (2014). Omega-3 Fatty Acid Supplementation on Lipid Profiles in Dialysis Patients: Meta-analysis. Arch. Med. Res..

[162] He L., Li M.-S., Lin M., Zhao T.-Y., Gao P. (2016). Effect of fish oil supplement in maintenance hemodialysis patients: A systematic review and meta-analysis of published randomized controlled trials. Eur. J. Clin. Pharmacol..

[163] Singh S. (2013). Hypertension in chronic kidney disease. Clin. Queries Nephrol..

[164] Miller E.R., Juraschek S.P., Anderson C.A., Guallar E., Henoch-Ryugo K., Charleston J., Turban S., Bennett M.R., Appel L.J. (2013). The Effects of *n*-3 Long-Chain Polyunsaturated Fatty Acid Supplementation on Biomarkers of Kidney Injury in Adults with Diabetes: Results of the GO-FISH trial. Diabetes Care.

[165] Miller P.E., Van Elswyk M., Alexander D.D. (2014). Long-Chain Omega-3 Fatty Acids Eicosapentaenoic Acid and Docosahexaenoic Acid and Blood Pressure: A Meta-Analysis of Randomized Controlled Trials. Am. J. Hypertens..

[166] Friedman A.N. (2010). Omega-3 Fatty Acid Supplementation in Advanced Kidney Disease. Semin. Dial..

[167] Friedman A., Moe S. (2006). Review of the Effects of Omega-3 Supplementation in Dialysis Patients. Clin. J. Am. Soc. Nephrol..

[168] Noori N., Dukkipati R., Kovesdy C.P., Sim J.J., Feroze U., Murali S.B., Bross R., Benner D., Kopple J.D., Kalantar-Zadeh K. (2011). Dietary Omega-3 Fatty Acid, Ratio of Omega-6 to Omega-3 Intake, Inflammation, and Survival in Long-term Hemodialysis Patients. Am. J. Kidney Dis..

[169] An W.S., Lee S.M., Son Y.K., Kim S.E., Kim K.H., Han J.Y., Bae H.R., Park Y. (2012). Effect of omega-3 fatty acids on the modification of erythrocyte membrane fatty acid content including oleic acid in peritoneal dialysis patients. Prostaglandins Leukot. Essent. Fat. Acids.

[170] Gharekhani A., Khatami M.-R., Dashti-khavidaki S., Razeghi E., Noorbala A.-A., Hashemi-nazari S.-S., Mansournia M.-A. (2014). The effect of omega-3 fatty acids on depressive symptoms and inflammatory markers in maintenance hemodialysis patients: A randomized, placebo-controlled clinical trial. Eur. J. Clin. Pharmacol..

[171] Hung A.M., Booker C., Ellis C.D., Siew E.D., Graves A.J., Shintani A., Abumrad N.N., Himmelfarb J., Ikizler T.A. (2015). Omega-3 fatty acids inhibit the up-regulation of endothelial chemokines in maintenance hemodialysis patients. Nephrol. Dial. Transplant..

[172] Bazeley J., Bieber B., Li Y., Morgenstern H., de Sequera P., Combe C., Yamamoto H., Gallagher M., Port F.K., Robinson B.M. (2011). C-Reactive Protein and Prediction of 1-Year Mortality in Prevalent Hemodialysis Patients. Clin. J. Am. Soc. Nephrol..

[173] Liu S.-H., Li Y.-J., Wu H.-H., Lee C.-C., Lin C.-Y., Weng C.-H., Chen Y.-C., Chang M.-Y., Hsu H.-H., Fang J.-T. (2014). High-Sensitivity C-Reactive Protein Predicts Mortality and Technique Failure in Peritoneal Dialysis Patients. PLoS ONE.

[174] Möllsten A.V., Dahlquist G.G., Stattin E.-L., Rudberg S. (2001). Higher Intakes of Fish Protein Are Related to a Lower Risk of Microalbuminuria in Young Swedish Type 1 Diabetic Patients. Diabetes Care.

[175] Miller E.R., Juraschek S.P., Appel L.J., Madala M., Anderson C.A., Bleys J., Guallar E. (2009). The effect of n–3 long-chain polyunsaturated fatty acid supplementation on urine protein excretion and kidney function: Meta-analysis of clinical trials. Am. J. Clin. Nutr..

[176] Chou H.H., Chiou Y.Y., Hung P.H., Chiang P.C., Wang S.T. (2012). Omega-3 Fatty Acids Ameliorate Proteinuria but Not Renal Function in IgA Nephropathy: A Meta-Analysis of Randomized Controlled Trials. Nephron Clin. Pract..

[177] Lee S.M., Chung S.H., Park Y., Park M.K., Son Y.K., Kim S.E., An W.S. (2015). Effect of Omega-3 Fatty Acid on the Fatty Acid Content of the Erythrocyte Membrane and Proteinuria in Patients with Diabetic Nephropathy. Int. J. Endocrinol..

[178] Shimizu H., Ohtani K.-I., Tanaka Y., Sato N., Mori M., Shimomura Y. (1995). Long-term effect of eicosapentaenoic acid ethyl (EPA-E) on albuminuria of non-insulin dependent diabetic patients. Diabetes Res. Clin. Pract..

[179] Madsen T., Christensen J.H., Svensson M., Witt P.M., Toft E., Schmidt E.B. (2011). Marine *n*-3 polyunsaturated fatty acids in patients with end-stage renal failure and in subjects without kidney disease: A comparative study. J. Ren. Nutr..

[180] Friedman A.N., Saha C., Watkins B.A. (2008). A Feasbility Study of Erythrocyte Long Chain Omega-3 Polyunsaturated Fatty Acid Content and Mortality Risk in Hemodialysis Patients. J. Ren. Nutr..

[181] Terashima Y., Hamazaki K., Itomura M., Tomita S., Kuroda M., Hirata H., Hamazaki T., Inadera H. (2014). Inverse association between docosahexaenoic acid and mortality in patients on hemodialysis during over 10 years. Hemodial. Int..

[182] Hamazaki K., Terashima Y., Itomura M., Sawazaki S., Inagaki H., Kuroda M., Tomita S., Hirata H., Inadera H., Hamazaki T. (2011). Docosahexaenoic Acid Is an Independent Predictor of All-Cause Mortality in Hemodialysis Patients. Am. J. Nephrol..

[183] Friedman A.N., Yu Z., Tabbey R., Denski C., Tamez H., Wenger J., Thadhani R., Li Y., Watkins B.A. (2013). Inverse relationship between long-chain *n*-3 fatty acids and risk of sudden cardiac death in patients starting hemodialysis. Kidney Int..

[184] Shoji T., Kakiya R., Hayashi T., Tsujimoto Y., Sonoda M., Shima H., Mori K., Fukumoto S., Tahara H., Shioi A. (2013). Serum *n*-3 and *n*-6 Polyunsaturated Fatty Acid Profile as an Independent Predictor of Cardiovascular Events in Hemodialysis Patients. Am. J. Kidney Dis..

[185] Metcalf R.G., James M.J., Gibson R.A., Edwards J.R., Stubberfield J., Stuklis R., Roberts-Thomson K., Young G.D., Cleland L.G. (2007). Effects of fish-oil supplementation on myocardial fatty acids in humans. Am. J. Clin. Nutr..

[186] Kutner N.G., Clow P.W., Zhang R., Aviles X. (2002). Association of fish intake and survival in a cohort of incident dialysis patients. Am. J. Kidney Dis..

[187] Inoue T., Okano K., Tsuruta Y., Tsuruta Y., Tsuchiya K., Akiba T., Nitta K. (2015). Eicosapentaenoic Acid (EPA) Decreases the All-Cause Mortality in Hemodialysis Patients. Intern. Med..

[188] Svensson M., Schmidt E.B., Jørgensen K.A., Christensen J.H. (2006). *N*-3 Fatty Acids as Secondary Prevention against Cardiovascular Events in Patients Who Undergo Chronic Hemodialysis: A Randomized, Placebo-Controlled Intervention Trial. Clin. J. Am. Soc. Nephrol..

[189] Zabel R., Ash S., King N., Bauer J. (2010). Adherence to Fish Oil Intervention in Patients with Chronic Kidney Disease. J. Ren. Nutr..

[190] Chowdhury R., Stevens S., Gorman D., Pan A., Warnakula S., Chowdhury S., Ward H., Johnson L., Crowe F., Hu F.B. (2012). Association between fish consumption, long chain omega 3 fatty acids, and risk of cerebrovascular disease: Systematic review and meta-analysis. BMJ.

[191] Visioli F., Risé P., Barassi M.C., Marangoni F., Galli C. (2003). Dietary intake of fish vs. formulations leads to higher plasma concentrations of *n*-3 fatty acids. Lipids.

[192] Bernstein A.M., Pan A., Rexrode K.M., Stampfer M., Hu F.B., Mozaffarian D., Willett W.C. (2012). Dietary Protein Sources and the Risk of Stroke in Men and Women. Stroke.

[193] Johnson R.J., Sanchez-Lozada L.G., Nakagawa T. (2010). The effect of fructose on renal biology and disease. J. Am. Soc. Nephrol..

[194] (2013). Singh AKab, Kari JAc: Metabolic syndrome and chronic kidney disease. Curr. Opin. Nephrol. Hypertens..

[195] Bomback A.S., Derebail V.K., Shoham D.A., Anderson C.A., Steffen L.M., Rosamond W.D., Kshirsagar A.V. (2010). Sugar-sweetened soda consumption, hyperuricemia, and kidney disease. Kidney Int..

[196] Nakayama T., Kosugi T., Gersch M., Connor T., Sanchez-Lozada L.G., Lanaspa M.A., Roncal C., Perez-Pozo S.E., Johnson R.J., Nakagawa T. (2010). Dietary fructose causes tubulointerstitial injury in the normal rat kidney. Am. J. Physiol. Ren. Physiol..

[197] Cirillo P., Gersch M.S., Mu W., Scherer P.M., Kim K.M., Gesualdo L., Henderson G.N., Johnson R.J., Sautin Y.Y. (2009). Ketohexokinase-Dependent Metabolism of Fructose Induces Proinflammatory Mediators in Proximal Tubular Cells. J. Am. Soc. Nephrol..

[198] Shoham D.A., Durazo-Arvizu R., Kramer H., Luke A., Vupputuri S., Kshirsagar A., Cooper R.S. (2008). Sugary soda consumption and albuminuria: Results from the National Health and Nutrition Examination Survey, 1999–2004. PLoS ONE.

[199] Choi J.W.J., Ford E.S., Gao X., Choi H.K. (2008). Sugar-sweetened soft drinks, diet soft drinks, and serum uric acid level: The third national health and nutrition examination survey. Arthritis Care Res..

[200] Gao X., Qi L., Qiao N., Choi H.K., Curhan G., Tucker K.L., Ascherio A. (2007). Intake of Added Sugar and Sugar-Sweetened Drink and Serum Uric Acid Concentration in US Men and Women. Hypertension.

[201] Saldana T.M., Basso O., Darden R., Sandler D.P. (2007). Carbonated beverages and chronic kidney disease. Epidemiology.

[202] Yuzbashian E., Asghari G., Mirmiran P., Zadeh-Vakili A., Azizi F. (2016). Sugar-sweetened beverage consumption and risk of incident chronic kidney disease: Tehran Lipid and Glucose Study. Nephrology.

[203] Lin J., Curhan G.C. (2011). Associations of Sugar and Artificially Sweetened Soda with Albuminuria and Kidney Function Decline in Women. Clin. J. Am. Soc. Nephrol..

[204] Cheungpasitporn W., Thongprayoon C., O’Corragain O.A., Edmonds P.J., Kittanamongkolchai W., Erickson S.B. (2014). Associations of sugar-sweetened and artificially sweetened soda with chronic kidney disease: A systematic review and meta-analysis. Nephrology.

[205] Karalius V.P., Shoham D.A. (2013). Dietary sugar and artificial sweetener intake and chronic kidney disease: A review. Adv. Chronic Kidney Dis..

[206] Gersch M.S., Mu W., Cirillo P., Reungjui S., Zhang L., Roncal C., Sautin Y.Y., Johnson R.J., Nakagawa T. (2007). Fructose, but not dextrose, accelerates the progression of chronic kidney disease. Am. J. Physiol. Ren. Physiol..

[207] Tapia E., Cristóbal M., García-Arroyo F.E., Soto V., Monroy-Sánchez F., Pacheco U., Lanaspa M.A., Roncal-Jiménez C.A., Cruz-Robles D., Ishimoto T. (2013). Synergistic effect of uricase blockade plus physiological amounts of fructose-glucose on glomerular hypertension and oxidative stress in rats. Am. J. Physiol. Ren. Physiol..

[208] Wang D.D., Sievenpiper J.L., de Souza R.J., Chiavaroli L., Ha V., Cozma A.I., Mirrahimi A., Matthew E.Y., Carleton A.J., Di Buono M. (2012). The effects of fructose intake on serum uric acid vary among controlled dietary trials. J. Nutr..

[209] Brymora A., Flisiński M., Johnson R.J., Goszka G., Stefańska A., Manitius J. (2012). Low-fructose diet lowers blood pressure and inflammation in patients with chronic kidney disease. Nephrol. Dial. Transplant..

[210] Poulsen M.W., Hedegaard R.V., Andersen J.M., de Courten B., Bügel S., Nielsen J., Skibsted L.H., Dragsted L.O. (2013). Advanced glycation endproducts in food and their effects on health. Food Chem. Toxicol..

[211] Penfold S.A., Coughlan M.T., Patel S.K., Srivastava P.M., Sourris K.C., Steer D., Webster D.E., Thomas M.C., MacIsaac R.J., Jerums G. (2010). Circulating high-molecular-weight RAGE ligands activate pathways implicated in the development of diabetic nephropathy. Kidney Int..

[212] Kratochvilová M., Zakiyanov O., Kalousová M., Kříha V., Zima T., Tesař V. (2011). Associations of Serum Levels of Advanced Glycation end Products with Nutrition Markers and Anemia in Patients with Chronic Kidney Disease. Ren. Fail..

[213] Nakamura T., Sato E., Fujiwara N., Kawagoe Y., Ueda Y., Suzuki T., Yamada S., Takeuchi M., Fukami K., Ueda S. (2009). Positive association of serum levels of advanced glycation end products and high mobility group box–1 with asymmetric dimethylarginine in nondiabetic chronic kidney disease patients. Metabolism.

[214] Henle T., Miyata T. (2003). Advanced glycation end products in uremia. Adv. Ren. Replace. Ther..

[215] Koschinsky T., He C.-J., Mitsuhashi T., Bucala R., Liu C., Buenting C., Heitmann K., Vlassara H. (1997). Orally absorbed reactive glycation products (glycotoxins): An environmental risk factor in diabetic nephropathy. Proc. Natl. Acad. Sci. USA.

[216] Uribarri J., Peppa M., Cai W., Goldberg T., Lu M., Baliga S., Vassalotti J.A., Vlassara H. (2003). Dietary glycotoxins correlate with circulating advanced glycation end product levels in renal failure patients. Am. J. Kidney Dis..

[217] Semba R.D., Fink J.C., Sun K., Windham B.G., Ferrucci L. (2010). Serum Carboxymethyl-lysine, a Dominant Advanced Glycation End Product, is Associated with Chronic Kidney Disease: The Baltimore Longitudinal Study of Aging. J. Ren. Nutr..

[218] D’Agati V., Schmidt A.M. (2010). RAGE and the pathogenesis of chronic kidney disease. Nat. Rev. Nephrol..

[219] Goldin A., Beckman J.A., Schmidt A.M., Creager M.A. (2006). Advanced glycation end products sparking the development of diabetic vascular injury. Circulation.

[220] Ott C., Jacobs K., Haucke E., Navarrete Santos A., Grune T., Simm A. (2014). Role of advanced glycation end products in cellular signaling. Redox Biol..

[221] Scivittaro V., Ganz M.B., Weiss M.F. (2000). AGEs induce oxidative stress and activate protein kinase C-βII in neonatal mesangial cells. Am. J. Physiol. Ren. Physiol..

[222] Li J., Schmidt A.M. (1997). Characterization and Functional Analysis of the Promoter of RAGE, the Receptor for Advanced Glycation End Products. J. Biol. Chem..

[223] Coughlan M.T., Thorburn D.R., Penfold S.A., Laskowski A., Harcourt B.E., Sourris K.C., Tan A.L., Fukami K., Thallas-Bonke V., Nawroth P.P. (2009). RAGE-induced cytosolic ROS promote mitochondrial superoxide generation in diabetes. J. Am. Soc. Nephrol..

[224] Hou F.F., Ren H., Owen W.F., Guo Z.J., Chen P.Y., Schmidt A.M., Miyata T., Zhang X. (2004). Enhanced Expression of Receptor for Advanced Glycation End Products in Chronic Kidney Disease. J. Am. Soc. Nephrol..

[225] Busch M., Franke S., Muller A., Wolf M., Gerth J., Ott U., Niwa T., Stein G. (2004). Potential cardiovascular risk factors in chronic kidney disease: AGEs, total homocysteine and metabolites, and the C-reactive protein. Kidney Int..

[226] Busch M., Franke S., Wolf G., Brandstädt A., Ott U., Gerth J., Hunsicker L.G., Stein G. (2006). The Advanced Glycation End Product Nε-Carboxymethyllysine Is Not a Predictor of Cardiovascular Events and Renal Outcomes in Patients With Type 2 Diabetic Kidney Disease and Hypertension. Am. J. Kidney Dis..

[227] Schwedler S.B., Metzger T., Schinzel R., Wanner C. (2002). Advanced glycation end products and mortality in hemodialysis patients. Kidney Int..

[228] Stein G., Busch M., Muller A., Wendt T., Franke C., Niwa T., Franke S. (2003). Are advanced glycation end products cardiovascular risk factors in patients with CRF?. Am. J. Kidney Dis..

[229] Suliman M.E., Heimburger O., Barany P., Anderstam B., Pecoits-Filho R., Rodriguez Ayala E., Qureshi A.R., Fehrman-Ekholm I., Lindholm B., Stenvinkel P. (2003). Plasma pentosidine is associated with inflammation and malnutrition in end-stage renal disease patients starting on dialysis therapy. J. Am. Soc. Nephrol..

[230] Wagner Z., Molnár M., Molnár G.A., Tamaskó M., Laczy B., Wagner L., Csiky B., Heidland A., Nagy J., Wittmann I. (2006). Serum Carboxymethyllysine Predicts Mortality in Hemodialysis Patients. Am. J. Kidney Dis..

[231] Meerwaldt R., Hartog J.W.L., Graaff R., Huisman R.J., Links T.P., den Hollander N.C., Thorpe S.R., Baynes J.W., Navis G., Gans R.O.B. (2005). Skin Autofluorescence, a Measure of Cumulative Metabolic Stress and Advanced Glycation End Products, Predicts Mortality in Hemodialysis Patients. J. Am. Soc. Nephrol..

[232] Feng J.X., Hou F.F., Liang M., Wang G.B., Zhang X., Li H.Y., Xie D., Tian J.W., Liu Z.Q. (2007). Restricted intake of dietary advanced glycation end products retards renal progression in the remnant kidney model. Kidney Int..

[233] Šebeková K., Faist V., Hofmann T., Schinzel R., Heidland A. (2003). Effects of a diet rich in advanced glycation end products in the rat remnant kidney model. Am. J. Kidney Dis..

[234] ŠEbeková K., Hofmann T., Boor P., ŠEbeková K., Ulicná O.G., Erbersdobler H.F., Baynes J.W., Thorpe S.R., Heidland A., Somoza V. (2005). Renal Effects of Oral Maillard Reaction Product Load in the Form of Bread Crusts in Healthy and Subtotally Nephrectomized Rats. Ann. N. Y. Acad. Sci..

[235] Zheng F., He C., Cai W., Hattori M., Steffes M., Vlassara H. (2002). Prevention of diabetic nephropathy in mice by a diet low in glycoxidation products. Diabetes Metab. Res. Rev..

[236] Somoza V., Lindenmeier M., Hofmann T., Frank O., Erbersdobler H.F., Baynes J.W., Thorpe S.R., Heidland A., Zill H., Bek S. (2005). Dietary bread crust advanced glycation end products bind to the receptor for AGEs in HEK-293 kidney cells but are rapidly excreted after oral administration to healthy and subtotally nephrectomized rats. Ann. N. Y. Acad. Sci..

[237] Tan A.L.Y., Sourris K.C., Harcourt B.E., Thallas-Bonke V., Penfold S., Andrikopoulos S., Thomas M.C., O’Brien R.C., Bierhaus A., Cooper M.E. (2010). Disparate effects on renal and oxidative parameters following RAGE deletion, AGE accumulation inhibition, or dietary AGE control in experimental diabetic nephropathy. Am. J. Physiol.-Ren. Physiol..

[238] Uribarri J., Peppa M., Cai W., Goldberg T., Lu M., He C., Vlassara H. (2003). Restriction of dietary glycotoxins reduces excessive advanced glycation end products in renal failure patients. Int. J. Am. Soc. Nephrol..

[239] Vlassara H., Cai W., Goodman S., Pyzik R., Yong A., Chen X., Zhu L., Neade T., Beeri M., Silverman J.M. (2009). Protection against loss of innate defenses in adulthood by low advanced glycation end products (AGE) intake: Role of the antiinflammatory age receptor-1. J. Clin. Endocrinol. Metab..

[240] Harcourt B.E., Sourris K.C., Coughlan M.T., Walker K.Z., Dougherty S.L., Andrikopoulos S., Morley A.L., Thallas-Bonke V., Chand V., Penfold S.A. (2011). Targeted reduction of advanced glycation improves renal function in obesity. Kidney Int..

